# Phylogenetic Clustering of Genes Reveals Shared Evolutionary Trajectories and Putative Gene Functions

**DOI:** 10.1093/gbe/evy178

**Published:** 2018-08-20

**Authors:** Chaoyue Liu, Benjamin Wright, Emma Allen-Vercoe, Hong Gu, Robert Beiko

**Affiliations:** 1 Faculty of Computer Science, Dalhousie University, Halifax, Nova Scotia, Canada; 2 Department of Mathematics and Statistics, Faculty of Mathematics and Statistics, Dalhousie University, Halifax, Nova Scotia, Canada; 3 Department of Molecular and Cellular Biology, University of Guelph, Ontario, Canada

**Keywords:** phylogenetic profiles, lateral gene transfer, microbial evolution, genomics

## Abstract

Homologous genes in prokaryotes can be described using phylogenetic profiles which summarize their patterns of presence or absence across a set of genomes. Phylogenetic profiles have been used for nearly twenty years to cluster genes based on measures such as the Euclidean distance between profile vectors. However, most approaches do not take into account the phylogenetic relationships amongst the profiled genomes, and overrepresentation of certain taxonomic groups (i.e., pathogenic species with many sequenced representatives) can skew the interpretation of profiles. We propose a new approach that uses a coevolutionary method defined by Pagel to account for the phylogenetic relationships amongst target organisms, and a hierarchical-clustering approach to define sets of genes with common distributions across the organisms. The clusters we obtain using our method show greater evidence of phylogenetic and functional clustering than a recently published approach based on hidden Markov models. Our clustering method identifies sets of amino-acid biosynthesis genes that constitute cohesive pathways, and motility/chemotaxis genes with common histories of descent and lateral gene transfer.

## Introduction

Lateral gene transfer (LGT) is an important force in microbial evolution, enabling processes such as adaptation to extreme environments (Ochman et al. 2000; Gogarten and Townsend 2005), acquisition of new metabolic functions (Pál et al. 2005), and defense against antimicrobial agents (Barlow 2009). Coordinated transfers of genes can enable rapid ecological shifts, and identification of sets of genes implicated in these shifts can highlight the events that took place during the diversification of prokaryotic groups, and suggest functional linkages between the implicated genes. Given the large phylogenetic diversity of microorganisms that inhabit the human microbiome (Turnbaugh et al. 2007), and in many cases the uncertainty associated with their precise ecological roles (Huttenhower et al. 2012), augmenting comparative genomic and metagenomic analysis with examination of LGT can produce more information about the capabilities of a given microorganism. Although strong evidence exists for preferential patterns or “highways” of gene sharing among specific groups of prokaryotes (Beiko et al. 2005; Skippington et al. 2011), small amounts of LGT connect many different taxonomic groups and the overall pattern of sharing resembles a web rather than a clear reticulated tree (Kloesges et al. 2011). These diffuse patterns, coupled with the methodological challenges associated with LGT inference (Philippe and Douady 2003; Kloesges et al. 2011), make it difficult to identify sets of genes with similar evolutionary trajectories.

Many approaches have been used to identify sets of genes with similar evolutionary histories. Puigbò et al. (2009) performed a comparative analysis of the topological similarity among 6,901 phylogenetic trees built for clusters of orthologous groups of proteins representing a set of 100 prokaryotes, showing that LGT did not obscure a significant central tendency so that a consistent phylogenetic signal still exists. Kunin et al. (2005) applied tree reconstruction methods to infer vertical evolutionary inheritance and then detect LGT events by using an ancestral-state inference algorithm and estimated the number of genes exchanged across organisms using a weighting scheme. The results suggest genes might propagate across a microbiome rapidly, with certain organisms functioning as hubs in a broader LGT network. Phylogenetic profiles (Pellegrini et al. 1999) summarize the presence and absence of homologous genes across a set of organisms, and have been used to identify laterally transferred genes (Ochman et al. 2000) and to predict the functions of hypothetical genes (Pellegrini 2012). Differences in gene content between related species result from processes such as gene loss, duplication and LGT, and proteins that are involved in similar biological processes (BPs) may be gained and lost together, leading to similar phylogenetic profiles. However, taxonomic sampling can pose a serious challenge to the interpretation of phylogenetic profiles. Calculating Manhattan or bit distances between pairs of profiles, for example, can be overly simplistic because it weights each contributing genome equally. In a scenario where the phylogenetic sampling of genomes is nonuniform, these distances will be unduly influenced by closely related genomes that have similar profiles due to common descent. The most informative profiles will be those that are widely but sporadically dispersed across very distantly related genomes, as their distribution will not be readily explained solely through a hypothesis of common descent. Vert (2002) and Barker and Pagel (2005) applied phylogenetic reweighting schemes to the assessment and comparison of phylogenetic profiles, and showed that genes that evolve in a correlated fashion also tend to be functionally linked. CLIME (Radivojac et al. 2013), short for Clustering by Inferred Models of Evolution, was developed to explicitly consider phylogenetic relationships among genes by inferring evolutionarily conserved modules (ECMs) using a Bayesian mixture model. Each ECM represents a tree-structured hidden Markov model (HMM) with a single gain branch and branch-specific gene loss probabilities. CLIME assigns genes within the genome to the most likely ECM or a new ECM by comparing with a background null model using the likelihood-ratio test. However, CLIME is based on models in which genes can emerge only once and then be lost multiple times. Since the effect of LGT is to create multiple apparent gene gains throughout a tree, the model of CLIME may not be appropriate for modelling prokaryotic genes that are subject to significant amounts of LGT.

**Fig. 1.**
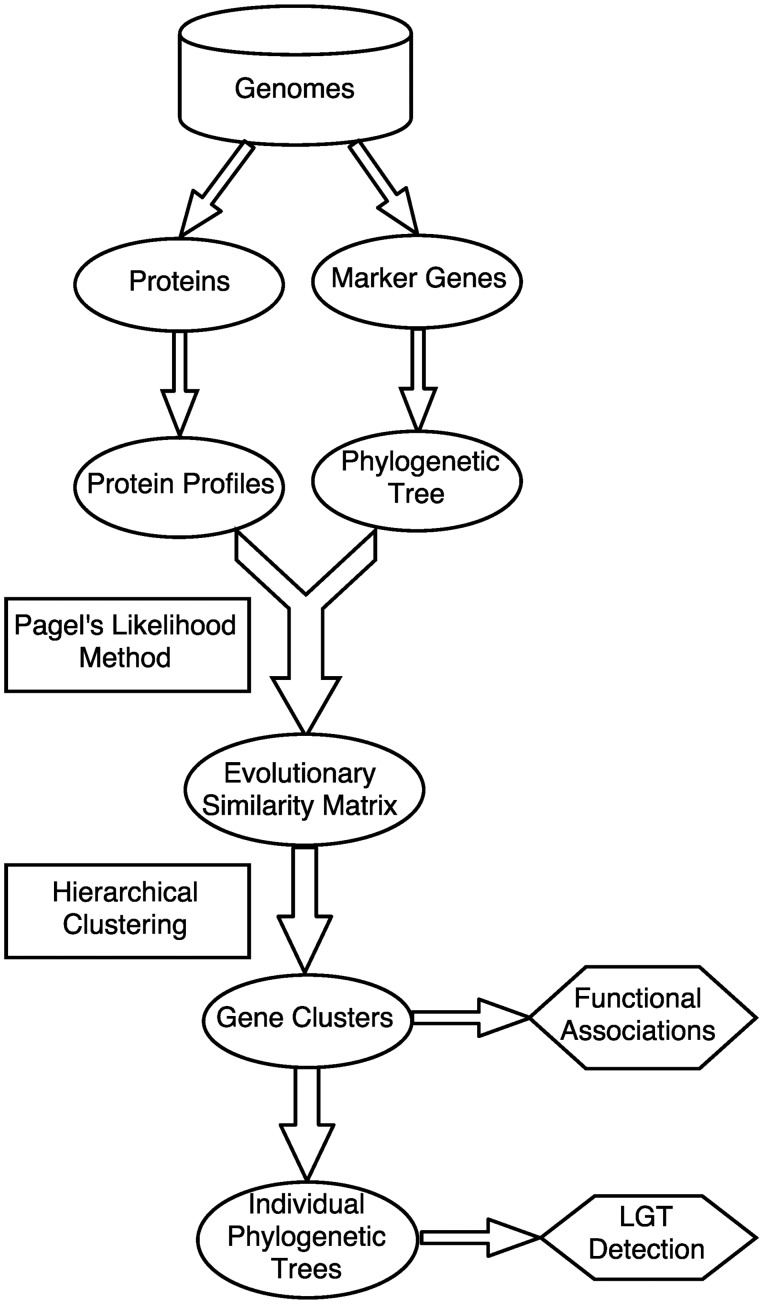
—Workflow of our weighted phylogenetic profile approach. Genome-sequence are collected to construct the protein profiles and phylogenetic tree. Then, Pagel’s likelihood method is implemented to calculate the evolutionary similarities among genes and the hierarchical-clustering approach is used to define sets of genes with common distributions across the given genomes. An evaluation framework based on the GO terms (BP) is also developed to study the function associations in the clustering results. In addition, the individual trees of the members within a same cluster are compared with detect potential LGT events.

Here, we propose a phylogenetic-profile-based method that uses phylogenetic modelling to identify pairs of genes with similar historical patterns of gain and loss. Our approach uses the method of Pagel (1994) to test hypotheses of correlated evolution between pairs of genes. The statistics generated by this approach are used to generate clusters using a hierarchical approach based on average linkage. The resulting clusters can be evaluated in terms of the similarity of their phylogenetic distributions, the functional similarity of the proteins in each cluster, and the phylogenetic trees built from different sets of proteins contained within the cluster, which can be further used to detect LGT events and infer genome evolution via tree reconstruction methods. The main work flow of this study is shown in figure 1.

One taxonomic group that has shown evidence for high levels of LGT is the class *Clostridia*. As part of the *Firmicutes* phylum, the class is a significant component of the human microbiome and contains an ecologically diversified set of organisms, including the pathogens *Clostridioides difficile* (previously *Clostridium difficile*; a notorious cause of nosocomial diarrhea), commensals from genera such as *Roseburia* and *Faecalibacterium*, and organisms that are less well understood (Pflughoeft and Versalovic 2012; Antharam et al. 2013). Commensurate with its ecological importance, over 1,000 genomes from class *Clostridia* have been sequenced at least in draft form, providing a rich resource for comparative genomics. Here, we apply our new method to a set of 687 genomes from class *Clostridia*, with a particular focus on *Lachnospiraceae* bacterium 3-1-57FAA-CT1, a microorganism which was isolated from a patient with Crohn’s disease and has an abnormally large genome for the group. Our method successfully recovers phylogenetically and functionally cohesive clusters of genes, and highlights probable highways of gene sharing that have shaped this genome and its close neighbors.

## Materials and Methods

### Data Sets

The bacterium “*Lachnospiraceae bacterium* 3-1-57FAA-CT1” lacks a formal taxonomic designation, and we refer to in this paper as “LachnoZilla” or LZ. LZ was isolated from a biopsy retrieved from the transverse colon of a female Crohn’s Disease patient aged 22 years at the time of colonoscopy. The patient was suffering a flare at the time of colonoscopy and biopsy material recovered was taken from an inflamed site. Isolation was carried out through serial dilution and culture on fastidious anaerobe agar (Acumedia) containing 5% defibrinated sheep’s blood (Hemostat Laboratories) with streak purification. gDNA was extracted using a Maxwell 16 instrument (Promega) according to manufacturer’s instructions. Sequencing was performed at the Broad Institute, as part of the Human Microbiome Project Reference Genomes effort (http://www.hmpdacc.org/reference_genomes/reference_genomes.php), generating sequence data to 140× coverage. The protein-coding genes were predicted with Prodigal (Hyatt et al. 2010) and filtered to remove genes with ≥70% overlap to tRNAs or rRNAs. The tRNAs were identified by tRNAscan-SE (Lowe and Eddy 1997). The rRNA genes were predicted using RNAmmer (Lagesen et al. 2007). The gene-product names were assigned based on top BLAST hits against the UniProtKB/SwissProt protein database (≥70% identity and ≥70% query coverage), and protein family profile search against the TIGRfam HMMer equivalogs.

We retrieved all available completed and draft genomes from class *Clostridia* (687 genomes including LZ; supplementary table S1, Supplementary Material online), and a set of eight outgroup genomes from class *Bacilli* and phyla *Actinobacteria* and *Proteobacteria* which are used to root the phylogenetic tree. All genome information used in this work were retrieved from the National Center for Biotechnology Information on August 22, 2014.

### Phylogenetic Analysis and Profile Construction

We used a customized version of the AMPHORA2 pipeline (Wu and Scott 2012) to construct a reference phylogeny based on concatenated, conserved protein sequences encoded by the set of genomes. Complete protein sequences were searched against the set of HMMs specified by AMPHORA2, yielding a maximum of 31 protein sequences per genome. Each set of homologous proteins was aligned using the corresponding HMM, then trimmed to remove any column that had a scaled alignment confidence score <7. Trimmed alignment files were then concatenated into a single alignment, with any missing genes represented in the alignment using missing-data (i.e., gap) characters. Maximum-likelihood phylogenetic analysis of this supermatrix was performed using RAxML-HPC version 7.2.5, using all default parameters and the “PROTCATLG” model of sequence substitution (Stamatakis 2006). One hundred bootstrap replicate alignments were generated using the SEQBOOT package of PHYLIP version 3.695, and the resulting bootstrap support values mapped to the appropriate bipartitions in the tree. The tree was rooted arbitrarily among the eight outgroup taxa, providing a defined rooting of the clostridial subtree.

Phylogenetic profiles were constructed using rapsearch version 2.14 (Ye et al. 2011). The complete set of predicted LZ proteins was compared against all other genomes in the data set, with an expectation-value threshold of 10^−20^. Profiles were interpreted as presence/absence matrices, with no weighting of profiles by the number of matching proteins in a given reference genome. Given the computational demands of the Pagel method, we uniformly subsampled 73 random taxa in addition to LZ from the full tree (supplementary fig. S1, Supplementary Material online), to produce a more tractable data set for cluster construction.

### Modelling Correlated Patterns of Evolution among Sets of Proteins

We used the BayesTraits software that implements the statistical approach of Pagel (1994) to correct profiles for shared evolutionary history. This method aims to identify significant evolutionary correlations between two discrete characters, which in our case corresponds to the presence or absence of two different homologous gene families, as represented by their phylogenetic profiles across a phylogenetic tree. To characterize the discrete-trait evolution in this method, two continuous-time Markov models are contrasted: One model where the two characters are assumed to evolve independently, and a second model where two characters are assumed to evolve in a correlated way, possibly due to interactions. The hypothesis of correlated evolution is tested by comparing the fit of the two different models to the observed data set. Under the assumption that the two characters evolve independently, the null model (independent evolution model) is a special case of the alternative model (dependent evolution model), and the two models can be assessed using a likelihood-ratio test. The dependent-evolution model with more parameters will almost certainly have a higher likelihood. Thus, the likelihood ratios, which follow a χ^2^ distribution with four degrees of freedom (difference in parameters), will express the relative strength of the evolutionary dependencies between genes.

We used the resulting likelihood ratios as the basis for a hierarchical clustering of all profiles. The likelihood ratios for all pairs of profiles were subtracted from the largest such ratio to generate a symmetrical 2,697 × 2,697 distance matrix. Clustering of this distance matrix was performed using the method of between-group average linkage (UPGMA). Specific clusters for analysis were generated by cutting the resulting dendrogram at different heights *h*.

### Evaluation of Profiles Based on Phylogenetic and Functional Similarity

Gene Ontology (GO) is a widely used classification scheme that was also used in the Critical Assessment of Functional Annotation (CAFA) large-scale evaluation experiment (Radivojac et al. 2013). To measure the performance of the clustering methods, we developed a framework based on the BP category from GO to evaluate the clustering results. All the available GO annotation of the proteins in this study are acquired from the Uniprot Knowledgebase (www.uniprot.org). To measure the biological significance, we evaluate the clusters from two directions: The quality of the clustering and the enrichment of GO terms. To evaluate the performance of the hierarchical clustering at different cutting heights, we calculate the mean of GO semantic scores weighted by the sizes of clusters according to the G-SESAME method (Wang et al. 2007) which accounts for the fraction of the aggregate contribution of all GO terms up to the closest shared ancestor term. In order to quantify the extent to which the clusters of co-evolved genes are functionally related, we performed a GO enrichment test for the distribution of each GO term across the clusters of co-evolved genes. We adopted the Pearson’s Chi-squared statistic as our test statistic. However, the chi-square distribution is not appropriate for this test because there are many gene clusters relative to the number of members in each GO term, which will result in many clusters with zero count of the considered GO term. To address this limitation, we used a resampling technique to estimate the null distribution of the Pearson’s Chi-squared statistic by randomly assigning 100,000 times all the GO terms to the clusters of genes with the sizes given by the sizes of our clusters of co-evolved genes from the hierarchical clustering methods. This test is similar to the hypergeometric tests but faster in computation.

## Results

### Genome Phylogeny and Profiles

**Fig. 2.**
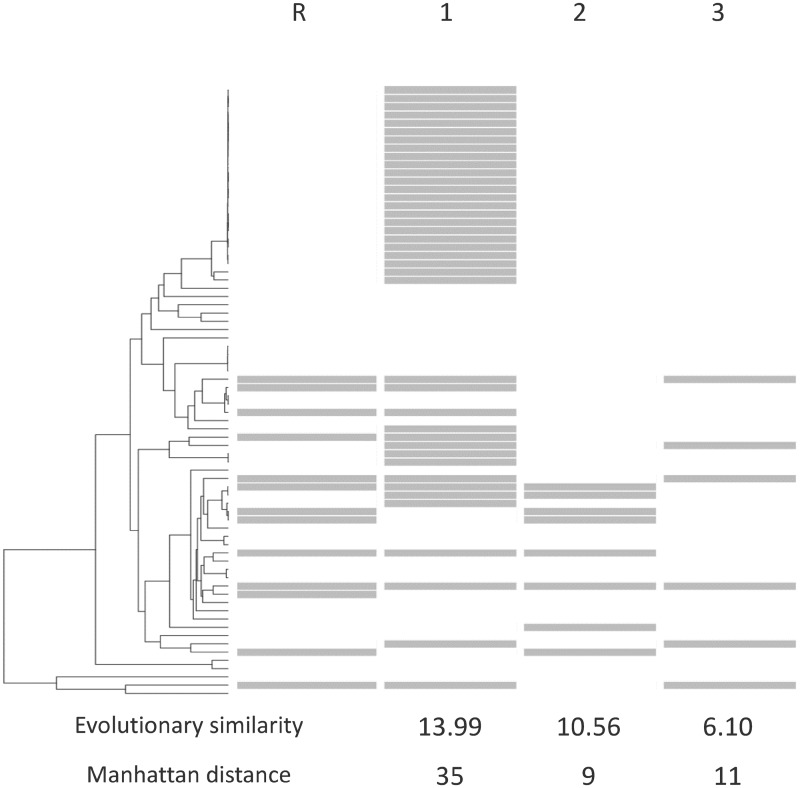
—Similarity of phylogenetic profiles (1, 2, 3) to a reference profile (R) according to Pagel’s likelihood-ratio statistics and Manhattan distance scores. Gray bars indicate the presence of a given gene in the genome that corresponds to the phylogenetic tree on the left. When considering the Manhattan distance, Gene 2 is the most similar to the reference profile, whereas Gene 1 has a very large Manhattan distance due to its representation in a closely related set of *Clostridioides difficile* genomes that do not contain genes in profile R. However, this drastic dissimilarity can be attributed to a single LGT event, and Gene 1 is most similar to the reference profile according to the likelihood-ratio statistic.

All profiles were constructed from a set of 687 genomes, including LZ (supplementary table S1, Supplementary Material online). A total of 21 genera were represented, with 38 genomes from genus *Clostridium* including 20 genomes of *C. difficile*. Seven genomes including LZ were not taxonomically assigned at the genus level, although the SILVA taxonomy (Quast et al. 2012; Yilmaz et al. 2014) assigned LZ to the genus *Eisenbergiella*. A total of 6,505 profiles were constructed with these genomes, including 2,814 proteins unique to LZ (supplementary fig. S2*a*, Supplementary Material online). A total of 2,697 distinct profiles were obtained (supplementary fig. S2*b*, Supplementary Material online) based on the uniformly subsampled 74 genomes. Figure 2 illustrates the differences between the nonphylogenetic Manhattan distance and Pagel’s likelihood based co-evolutionary method in contrasting the similarity of three phylogenetic profiles to a reference profile.

### Robustness of Pagel’s Statistics

Different tree rootings and the randomness in computing the maximum-likelihood estimators in Pagel’s software may affect the calculation of coevolutionary similarities, which can result in inconsistent likelihood-ratio statistics for the same pair of genes. To evaluate this instability, we reran the full data set using the other two tree-rooting methods: MAD which is based on the minimum ancestor deviation (Tria et al. 2017), and the naïve midpoint-rooting method. The likelihood-ratio statistics computed from three different ways of tree-rooting all showed correlation scores > 0.9 (supplementary fig. S4*a*–*c*, Supplementary Material online). The correlations are high despite the instability introduced by the errors involved in the maximum-likelihood computation process. We can conclude from these results that Pagel’s statistics are robust relative to different tree-rooting methods and the errors introduced in the computation of MLEs.

### Properties of Clusters Generated by the Hierarchical Method and CLIME

**Fig. 3.**
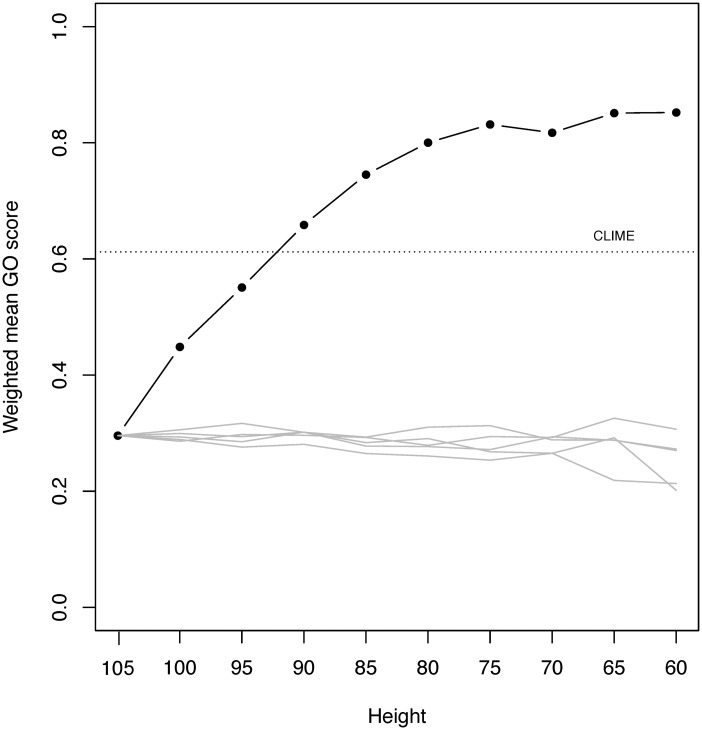
—Comparison of functional-similarity scores within clusters. The similarity was evaluated using Gene Ontology (GO) terms, for CLIME (dashed line) and the hierarchical approach at different *h* thresholds (solid line). Gray lines show the distribution of similarities obtained for five sets of clusters obtained by randomly reassigning tip labels in the cluster tree.

In spite of the similar cluster-size distributions produced by our method and CLIME, there are substantial differences in the clusters produced. We first used the weighted average of the GO semantic similarity to compare the overall clustering results between two methods. The CLIME approach, which generates a single set of clusters, yielded an average GO similarity within clusters of 0.61 (fig. 3). The hierarchical approach generated a large range of similarity values depending on the choice of threshold, from 0.3 when the cutting height *h* = 100, to 0.85 when *h* = 60. Both approaches generated similarity scores that were greater than random. The increase in GO similarity with decreasing *h* is reasonable, since lower values of *h* produce clusters with higher overall profile similarity. However, the cost of lower *h* is that fewer profiles are assigned to nonsingleton clusters.

A key distinction between the hierarchical approach and CLIME is the treatment of gene gain-and-loss events. CLIME allows only a single gain of a trait on the phylogenetic tree. In cases where a gene has a sparse distribution due to LGT, the CLIME model will be a poor fit. This is illustrated in supplementary figure S5, Supplementary Material online, where a hierarchical cluster containing four profiles (supplementary fig. S5*a*, Supplementary Material online), all annotated with the mannose metabolic (alpha-mannosidase activity) GO functional category, is split into four singleton clusters by CLIME. In spite of their similar phylogenetic distribution, CLIME’s single-gain constraint assigns events to different parts of the tree (supplementary fig. S5*b*–*e*, Supplementary Material online), yielding different historical inferences for these four profiles. We also compared with another novel probabilistic evolutionary model CoPAP (Cohen et al. 2012, 2013) at different cut-offs (supplementary fig. S6, Supplementary Material online) and showed that our method performs significantly better than other two methods (supplementary fig. S7 and table S5, Supplementary Material online) via a permutation test. However, the runtime of CoPAP was considerably faster than CLIME and our hierarchical method.

The most time-consuming step of our method is calculating the Pagel statistics. Each pairwise comparison of genes requires only a few seconds, but over 3 million comparisons are needed to do comparisons over all 2,600 genes. However, this step is easy to run in parallel because the pairs of genes are independent. In our case, we spent ∼7 days by running 30 jobs in parallel. For the same data set, CoPAP took around 3 h by using the web service developed by its authors and CLIME took around 1 day by running on a local computer.

### Biological Significance

**Fig. 4.**
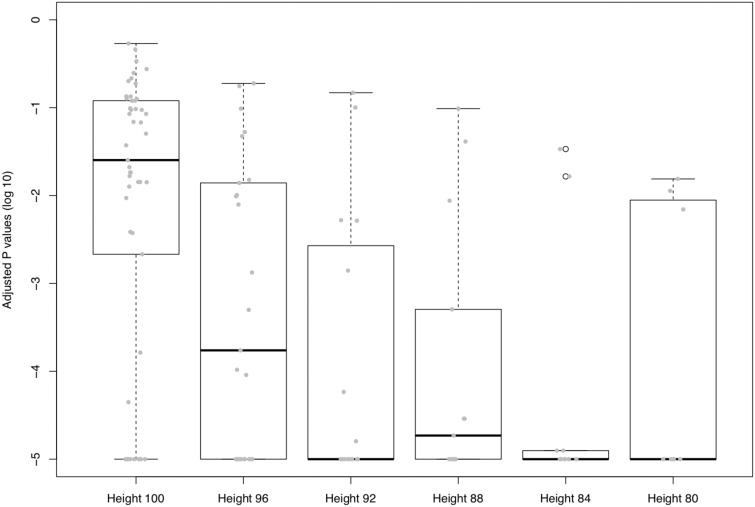
—Adjusted *P* values of the nonrare GO terms obtained at different cutting heights of the hierarchical dendrogram. Each dot represents a GO term with frequency > 5 in our gene set at different cutting heights. More details of the GO enhancement test are provided in supplementary table S2, Supplementary Material online.

To test the biological significance of our clustering results, we implemented a GO enrichment test by comparing the Pearson’s chi square statistics between the observed gene clusters and 100,000 randomly generated clusters in the same size distributions. We used the Benjamini–Hochberg FDR procedure to control for multiple tests. Figure 4 shows the significance of the nonrare GO terms (frequency ≥ 5) in our gene set at different cutting heights of the hierarchical dendrogram (the details of the test provided in supplementary table S2, Supplementary Material online). Among those interesting gene clusters, we take the amino-acid biosynthesis and motility-associated gene clusters as illustrative examples.

### Pathway Mapping of an Amino-Acid Biosynthesis Cluster

**Fig. 5.**
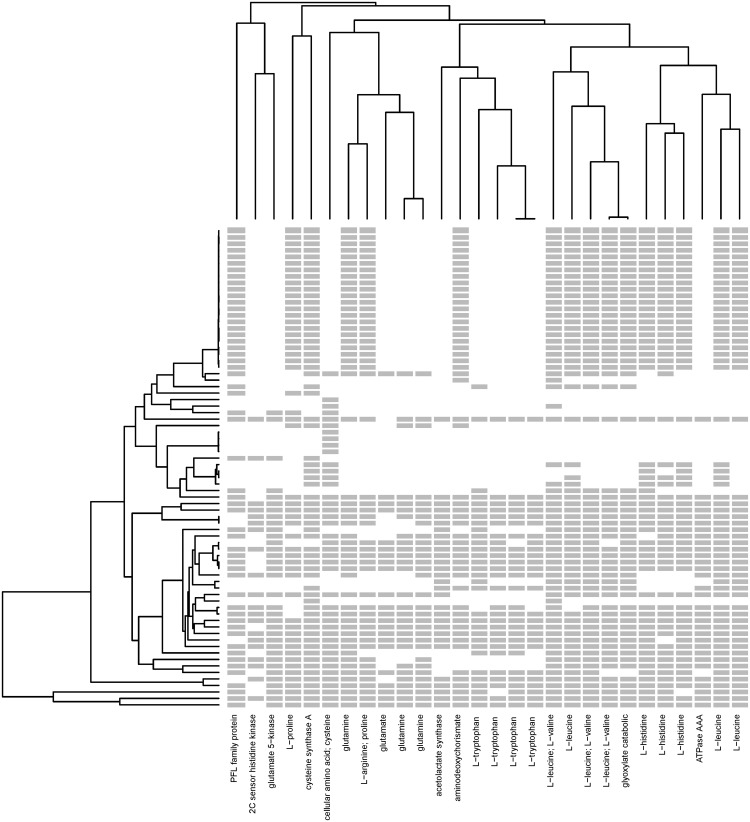
—Structure, phylogenetic distribution and functional categories of a hierarchical cluster enriched in amino-acid biosynthesis proteins. Each column represents a gene profile; the gray bars indicate the presence of genes and blanks indicate the absence. The dendrogram on the left side is the phylogenetic tree of 74 genomes and the dendrogram on the top is computed by the distance between the profiles. The labels on the *x*-axis are the genes’ functional annotations retrieved from the UniProt database.

From figure 3, it is anticipated that many clusters will have a high degree of functional cohesion based on the GO scores. However, examination of clusters with even relatively low GO similarity scores still showed strong functional similarities in spite of annotations with different terms. We examined in detail a cluster containing 28 distinct profiles, with a height of 100 and a GO score of 0.62 (fig. 5). A striking property of this cluster is that it contains many profiles that either include or exclude all of the 20 *C. difficile* genomes, whereas a phylogenetically naïve approach would assign a great deal of significance to this difference. Although the GO terms in this cluster are not identical, the majority of profiles assigned to the cluster are associated with amino-acid biosynthesis. Several amino-acid biosynthesis pathways are represented, including leucine, isoleucine, histidine, valine, tryptophan, glutamate/glutamine, and cysteine. Many of these pathways are tightly interconnected, notably valine and leucine, but some pathways, in particular histidine, are more distant. Supplementary figure S9, Supplementary Material online shows how the proteins in this cluster connect in the corresponding “*Valine, Leucine and Isoleucine biosynthesis*” and “*Phenylalanine, Tyrosine and Tryptophan biosynthesis*” pathways (KEGG database).

### Phylogenetic Analysis of a Motility-Associated Cluster

**Fig. 6.**
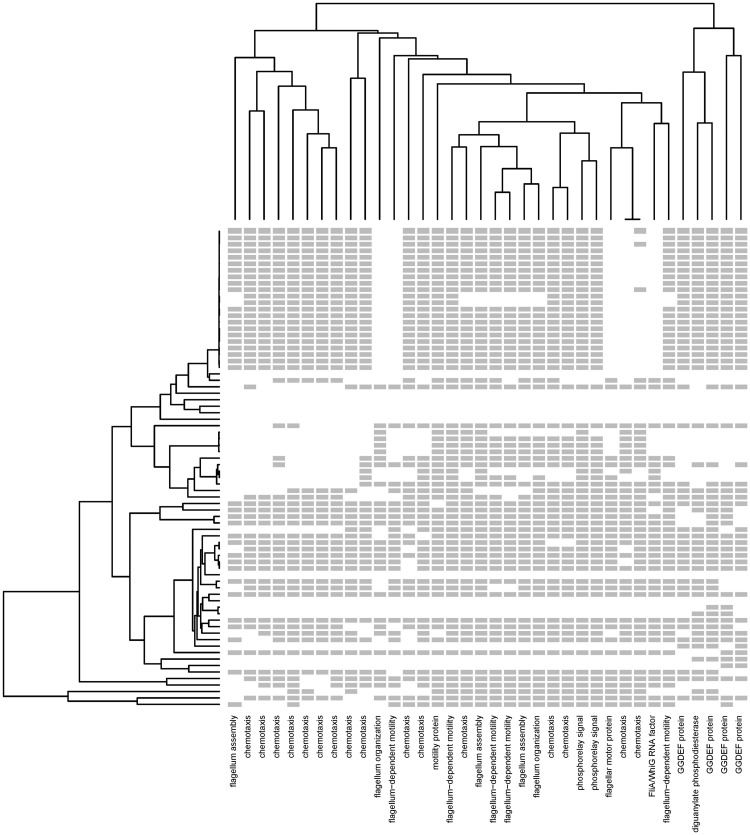
—Structure, phylogenetic distribution and functional categories of a hierarchical cluster enriched in flagellar motility proteins. Each column represents a gene profile; the gray bars indicate the presence of genes and blanks indicate the absence. The dendrogram on the left side is the phylogenetic tree of 74 genomes and the dendrogram on the top is computed by the distance between the profiles. The labels on the *x*-axis are the genes’ functional annotations retrieved from the UniProt database.

Figure 6 shows a functionally cohesive cluster consisting of the proteins related to flagellar assembly and motility, and their corresponding phylogenetic profiles. Besides predicting the functions for unannotated genes in this cluster, we can also infer the LGT events based on the evolutionary pattern we found. The patchy distribution of flagellar gene profiles supports a history that includes many LGT events. To assess the phylogenetic cohesion of the genes in this cluster, we constructed individual phylogenetic trees and compared them with the reference full tree. All flagellar proteins from LZ grouped with the almost same set of other genomes (supplementary table S3, Supplementary Material online): *Clostridium hylemonae* DSM15053, *Clostridiales bacterium* VE202-28, *C. bacterium* 1_7_47FAA, *Clostridium bolteae* 90B7, *C. bolteae* 90B8, none of which was a close neighbor in the reference tree or the SILVA taxonomy (supplementary table S1, Supplementary Material online).

### Connections between LZ and *C. bolteae*

LZ has a large genome containing 6,887 protein-coding genes, whereas the median genome size in our data set is 3,728 genes, and the mean genome size is 3580.4. One possible explanation for this disparity is a genome expansion due to gene duplication and LGT. The two strains of *C. bolteae* whose flagellar genes were proximal to those of LZ may show similar evidence of LGT with LZ and its close relatives. To address this possibility, we examined the homology-search results of LZ versus all genomes, and defined criteria to represent “unexpected similarity” between LZ proteins and their corresponding homologs in *C. bolteae.* We set threshold criteria that required the e-values of the match between the LZ protein and the *C. bolteae* protein sequence be <10^−20^, and also required that the *C. bolteae* protein rank within the top 20 of all matches from a given LZ protein to the entire database of 687 genomes. We then identified the corresponding profiles in our cluster tree, and implemented a binomial test to identify clusters in which these proteins were significantly overrepresented.

Individual profiles of proteins belonging to the flagellar-associated cluster were subjected to phylogenetic analysis. Each of the 36 profiles was first aligned using MUSCLE version 3.7 (Edgar 2004) with default parameters. The resulting alignments were used to infer phylogenetic trees using RAxML version 7.2.5, with the PROTGAMMALG model of sequence substitution. Using this approach, we identified the flagellum/motility cluster and three additional clusters. Although each of these clusters showed significant overrepresentation of the identified proteins, none was completely homogeneous in this regard: However, a majority of profiles did contain representatives from at least one of the two *C. bolteae* genomes (strain 90B7, 97.8%; strain 90B8, 89.6%). The predominant GO annotations within the three additional identified clusters comprised 1) relaxase/mobilization nuclease and ParB-like partition proteins (supplementary fig. S8*a*, Supplementary Material online); 2) sequence-specific DNA binding, ATPase activity, and methyltransferase activity (supplementary fig. S8*b*, Supplementary Material online); and 3) PAS domain S-box protein, two-component system hybrid sensor kinases, response regulators, and diguanylate cyclase (GGDEF) domain-containing proteins (supplementary fig. S8*c*, Supplementary Material online). Each cluster covers different subsets of the sampled genomes, but in each case, it is difficult to explain the distributions with only gene-loss events, suggesting an important role for LGT. Many of the functional annotations of these proteins are very general, but cluster 1) suggests a possible role for plasmid-based transfer, and 2) suggests that environmental sensitivity and response may be adaptive in a niche occupied by LZ (and/or *C. bolteae*).

## Discussion

Phylogenetic profiles were initially developed at a time when the relatively few sequenced genomes available were phylogenetically very diverse, and their similarity due to common descent was not explicitly incorporated into profile-similarity calculations. However, the intensive focus on sequencing many strains of some named species, notably those species that contain pathogenic isolates, has led to highly uneven sampling across the breadth of microbial diversity. An example in our data set is the >100 sequenced genomes of *C. difficile*, of which 21 were retained in our subsampled data set. Our new phylogenetic-profile-based clustering approach successfully addresses these phylogenetic correlations using Pagel’s method for the comparative analysis of discrete characters. The success of our approach is most striking in the many instances we show where clustered profiles can differ in the presence or absence of all 21 strains of *C. difficile*, whereas a phylogenetically naïve approach would assign a very large distance between such profiles. Furthermore, by explicitly allowing multiple gains of proteins in the tree rather than a single common ancestor followed by potentially many gene losses, our method is more suitable than CLIME in the analysis of LGT-prone prokaryotic genomes.

LZ has a very large genome relative to most other clostridia, and elucidating its ecological role will be challenging. However, by examining a subset of its clusters, we can identify not only specific functions that appear to be present in the genome of LZ, but also identify sets of proteins with similar (but not identical) distributions. Our analysis of even a small subset of LZ clusters shows a complex set of relationships with other genomes, but also highlights the functional cohesion of our recovered clusters. In the cases of amino-acid metabolism and flagellar/motility genes, complementary evidence from pathway diagrams, genetic linkage, and phylogenetic trees supports our inferred connections. We were also able to focus on a small subset of clusters in which LZ appeared to have unusual patterns of similarity to *C. bolteae*; the identified genes provided clues to transfer mechanisms, environmental adaptation, and potentially (in the case of flagella) pathogenicity (Stecher et al. 2012).

Protein functional prediction is one of the greatest challenges in bioinformatics (Sjolander 2004; Friedberg 2006; Radivojac et al. 2013). Although we did not explore the effectiveness of this method in functional prediction of proteins, the functional cohesion of many of our recovered clusters suggests that it may have value as a predictive tool. Since we use techniques that are based only indirectly on homology search, it may prove to be complementary to homology-based (PSI-BLAST) and other approaches.

Since more genomes provide more opportunities to differentiate profiles and give further resolution to clusters, a method that can consider all available genomes would be desirable. One significant limitation of our method is the heavy computational cost of applying Pagel’s coevolutionary method to all pairs of distinct phylogenetic profiles: Although our full data set included 687 genomes, computational time limitations restricted us to the analysis of a set of 74 genomes. Even this reduced computation required a total of 13,000 CPU hours approximately on a Linux system. Our future work will consider alternatives to Pagel’s method including phylogenetic regression and HMMs that also take phylogenetic correlations into account, and heuristics to subdivide the full tree into tractable subsets of taxa to perform the analysis, then merge the results to obtain a full set of distances. However, our results on even a small subset of available genomes demonstrate that our phylogenetic-profile-based clustering method has the capacity to identify sets of genes with similar distributions and evolutionary histories, with the potential to represent genomes as distinct combinations of these sets, thereby highlighting the important genetic and environmental connections between them.

## Supplementary Material

Supplementary data are available at *Genome Biology and Evolution* online.

## Supplementary Material

Supplementary Data
