# Supplementary material for: Phylogenetic Clustering of Genes Reveals Shared Evolutionary Trajectories and Putative Gene Functions
Source: Genome Biol Evol. 2018 Aug 20;10(9):2255–65. doi: 10.1093/gbe/evy178 (PMC6130602; doi:10.1093/gbe/evy178)
Supplement: Supplementary Data [file evy178_supp.zip › Figures_Supplementary.pdf]

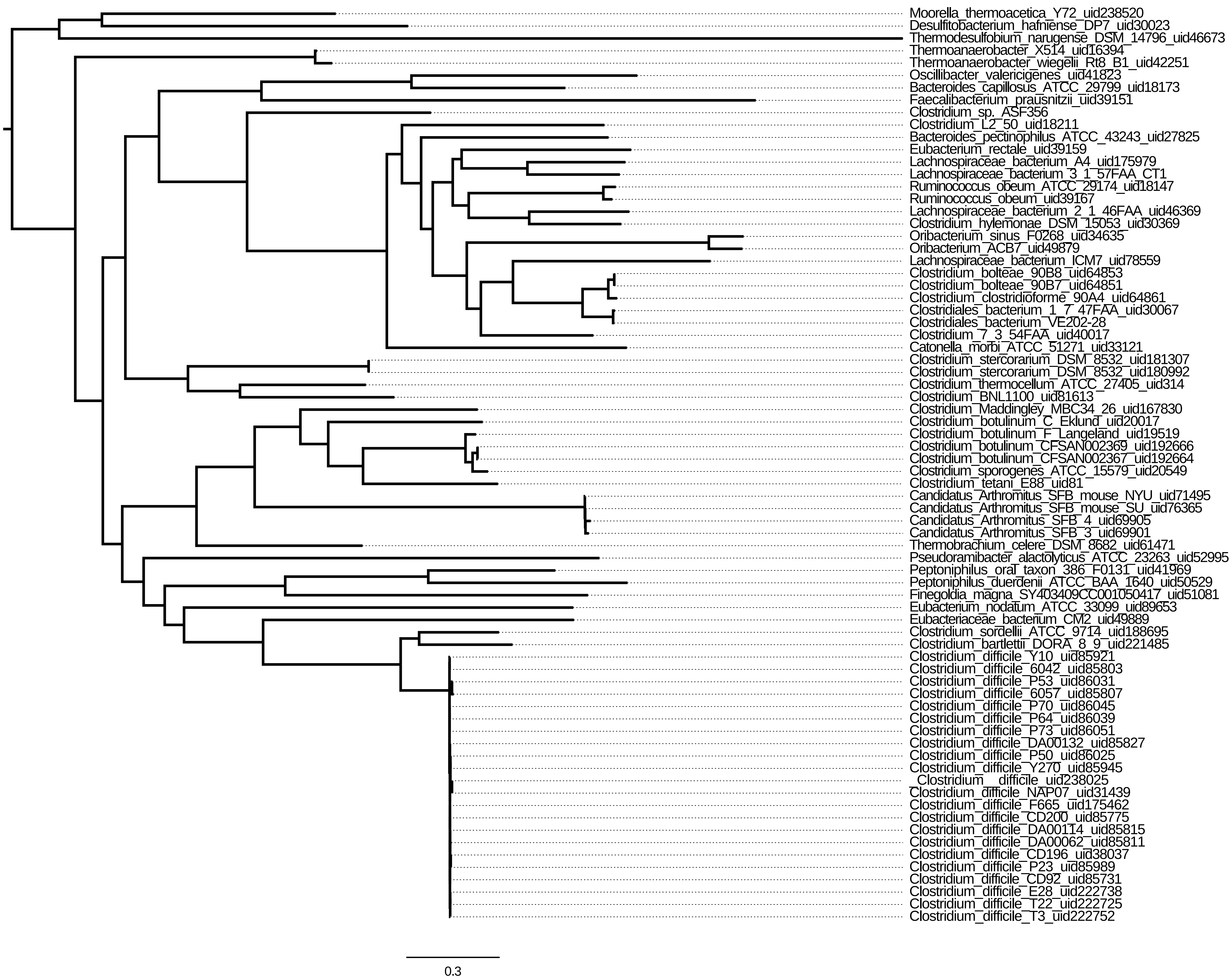

Figure S1. Phylogenetic tree of 74 genomes used to build profiles, subsampled from the full tree of 687 genomes.

(a)

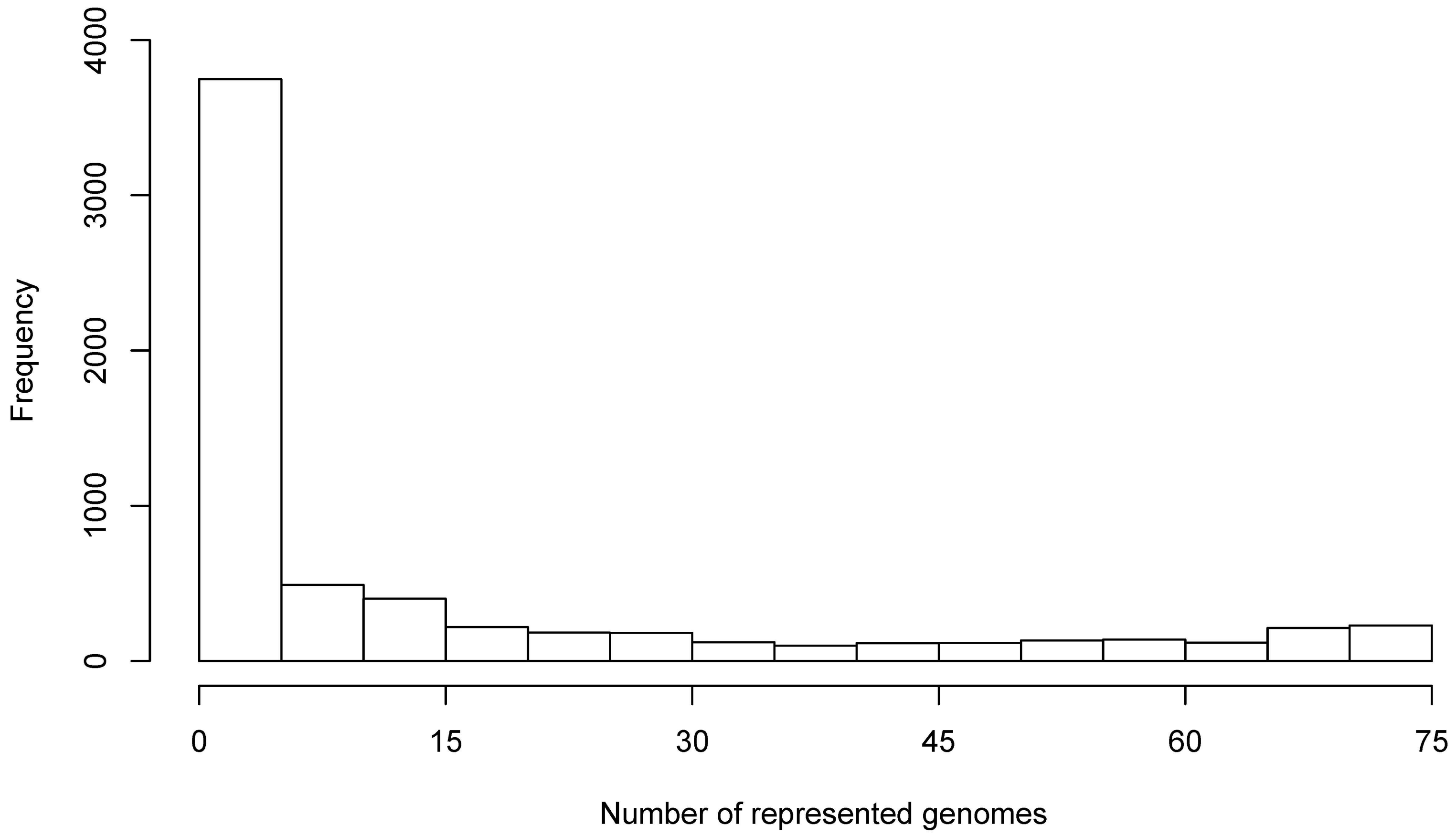

(b)

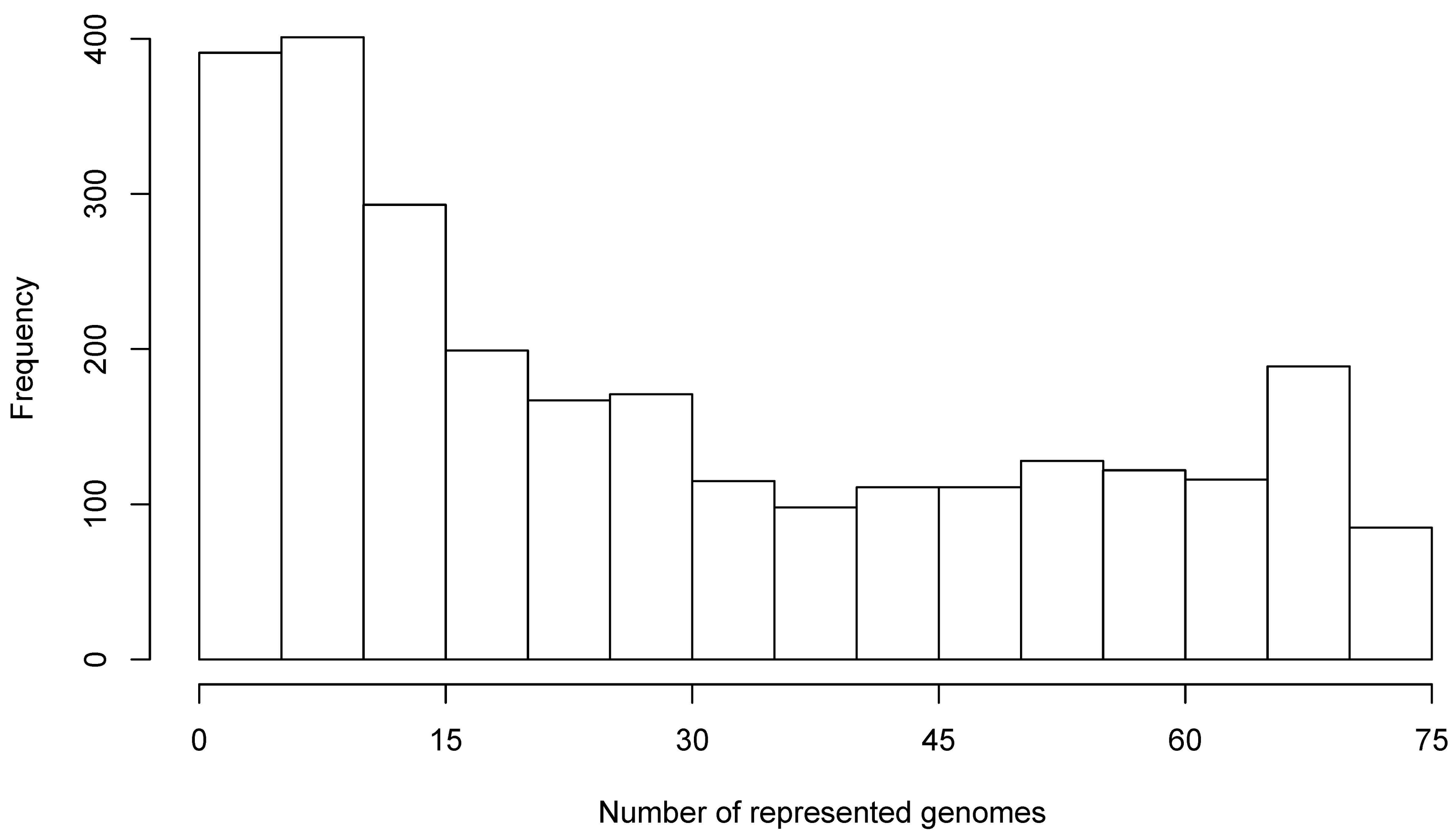

Figure S2. (a) Size distribution (number of presences) of 6505 profiles. (b) Size distribution (number of presences) of 2697 unique profiles.

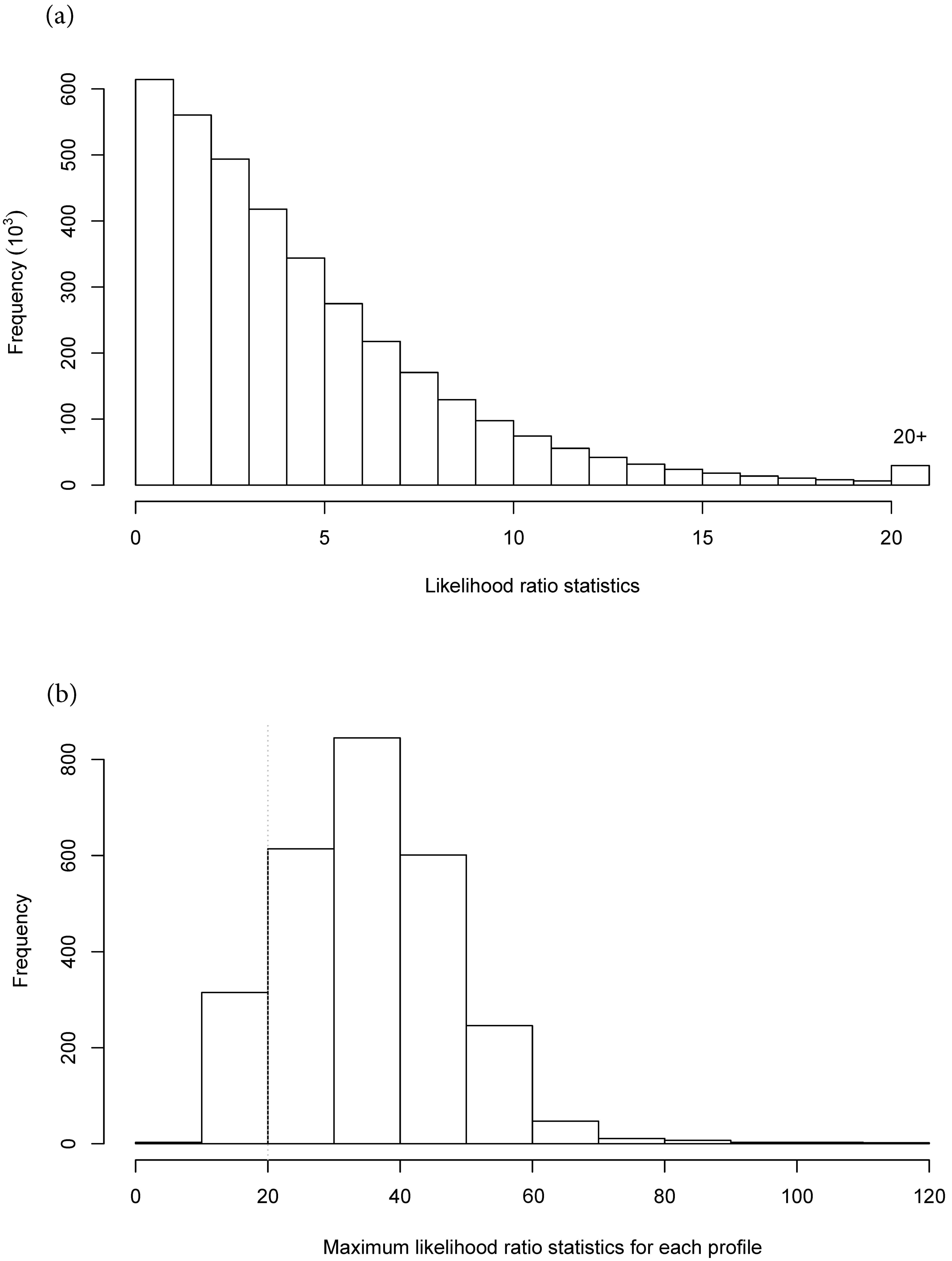

Figure S3. (a) Distribution of likelihood-ratio scores calculated from all pairs of phylogenetic profiles. (b) Maximum of all likelihood-ratio scores calculated for each phylogenetic profile in comparison with all other profiles.

(a)

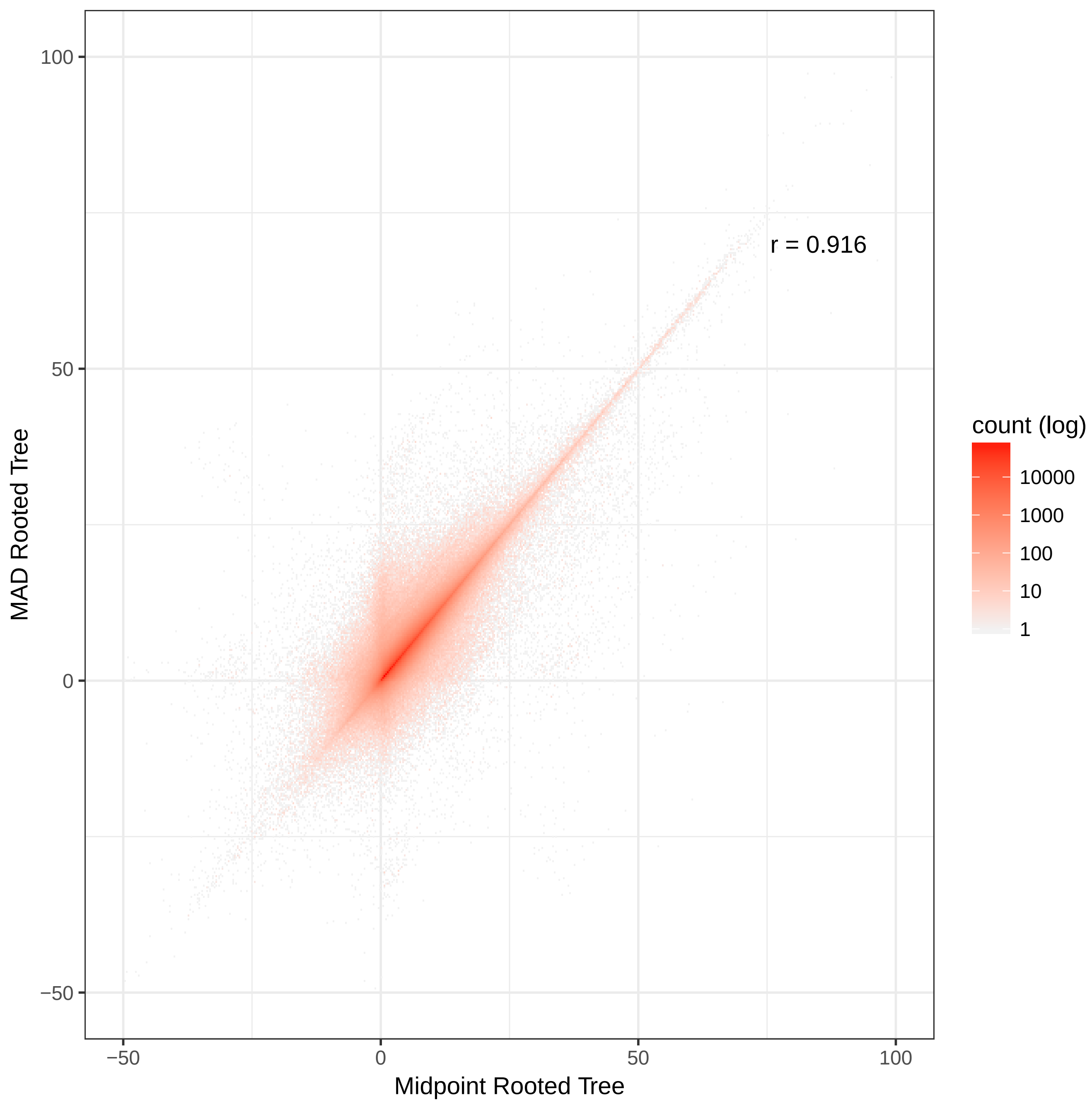

r = 0.916
MAD Rooted Tree
Midpoint Rooted Tree
count (log)
10000
1000
100
10
1
100
50
0
−50
−50
0
50
100

(b)

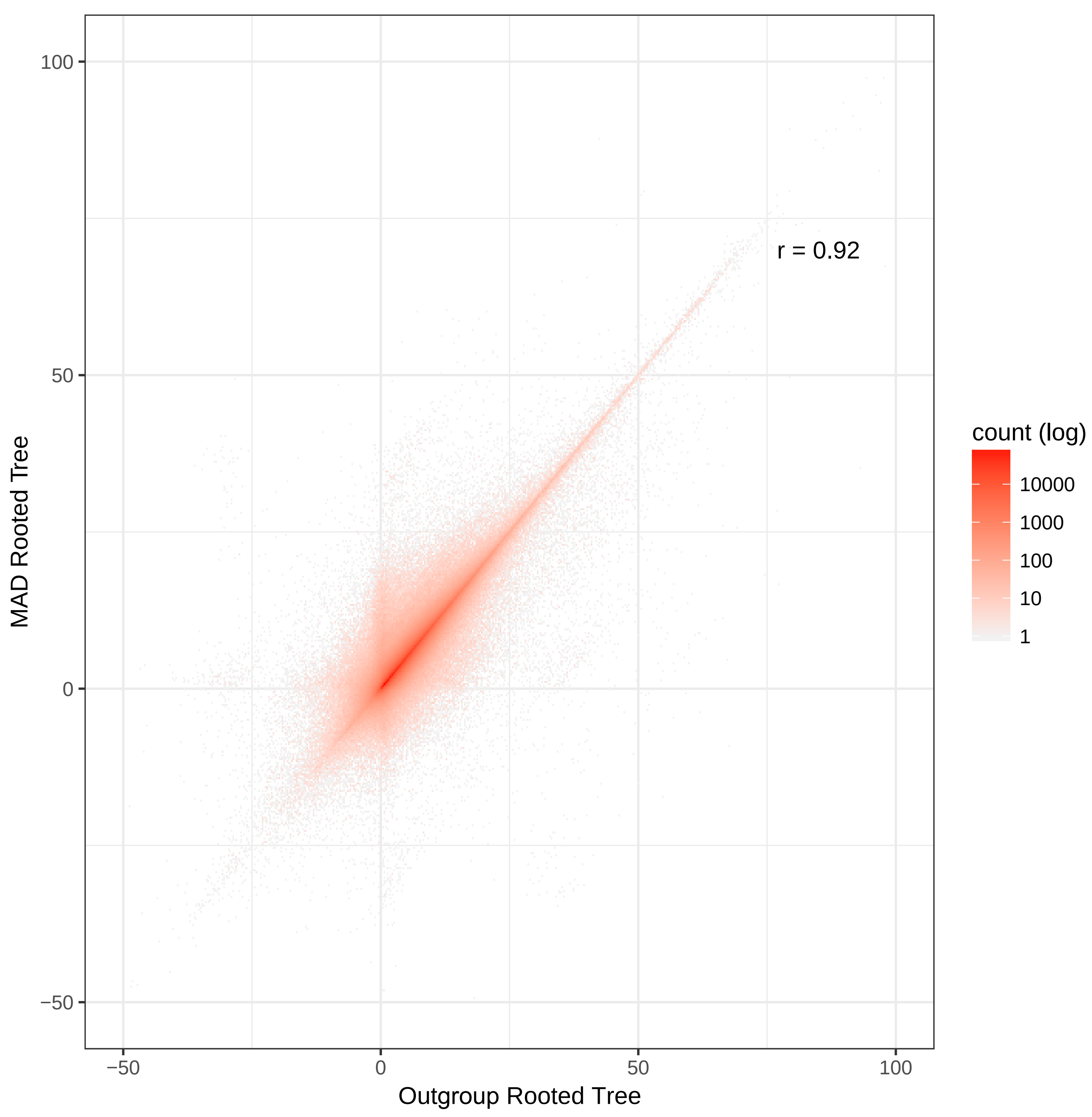

100
50
0
−50
MAD Rooted Tree
r = 0.92
−50
0
50
100
Outgroup Rooted Tree
count (log)
10000
1000
100
10
1

(c)

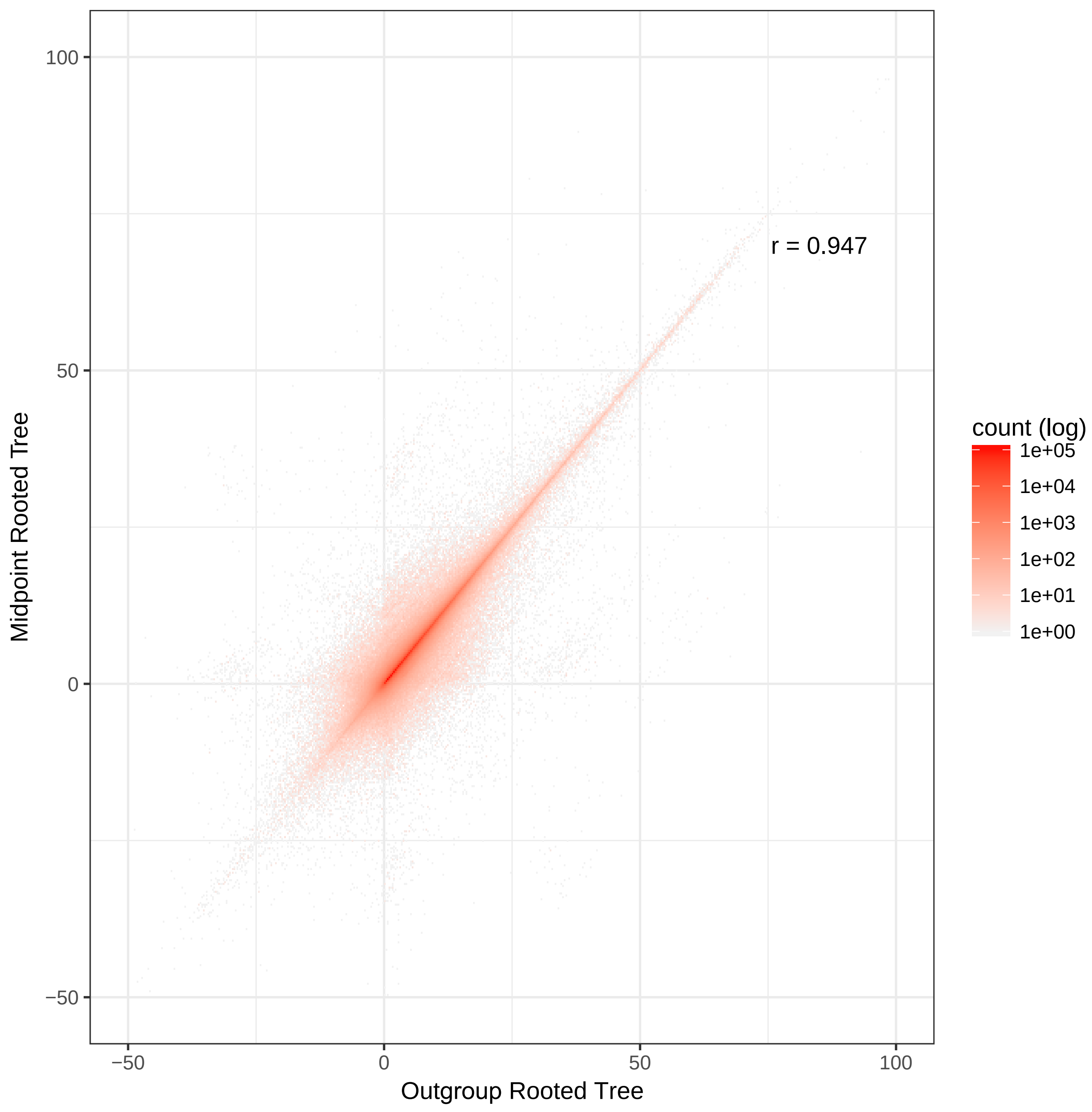

Figure S4. The comparisons of the Pagel's likelihood ratio statistics computed from three different ways of tree rooting: (a) Midpoint-rooted tree vs MAD-rooted tree; (b) Outgroup-rooted tree vs MAD-rooted tree; (c) Outgroup-rooted tree vs Midpoint-rooted tree.

(a)

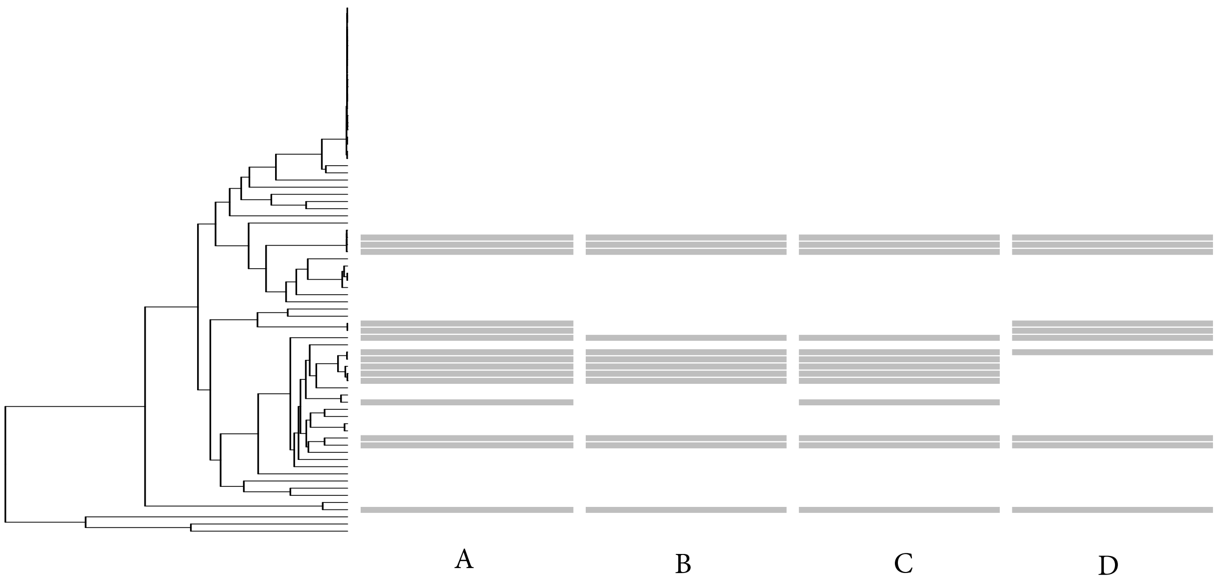

(b)

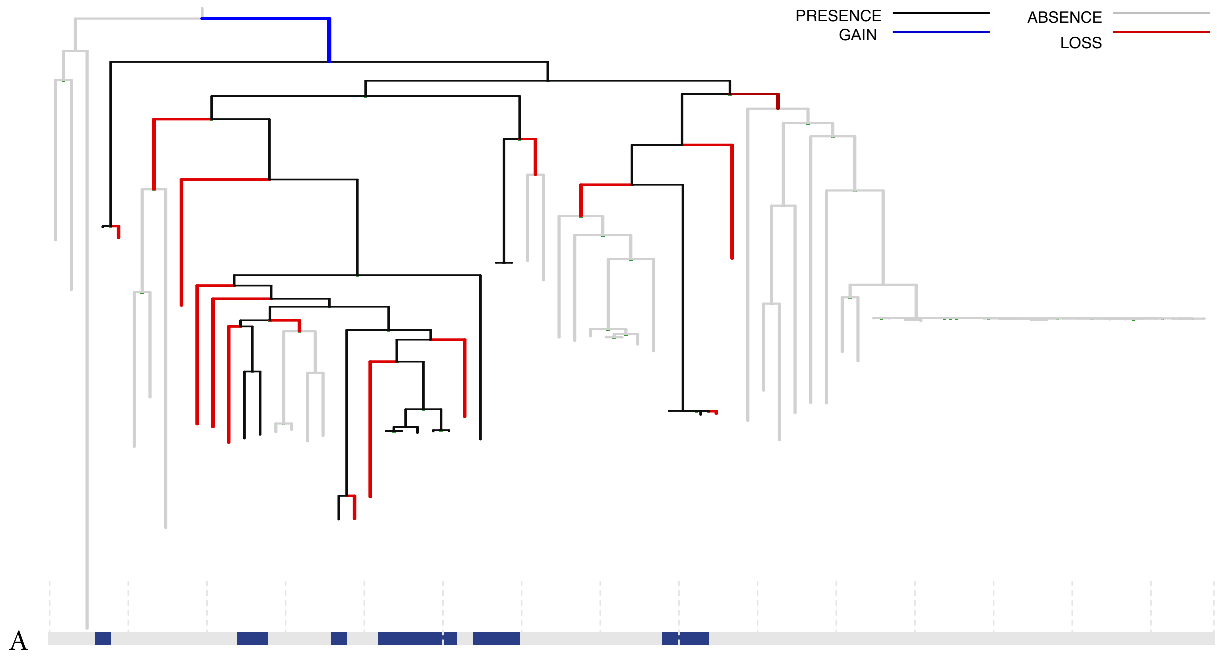

(c)

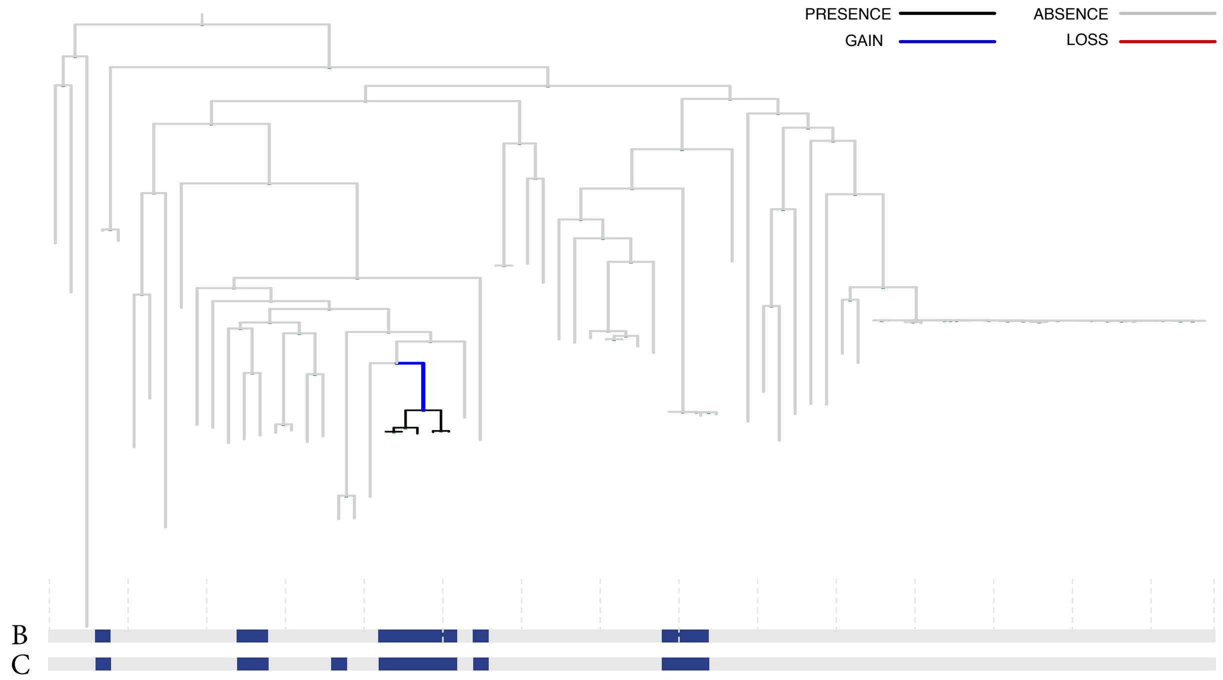

(d)

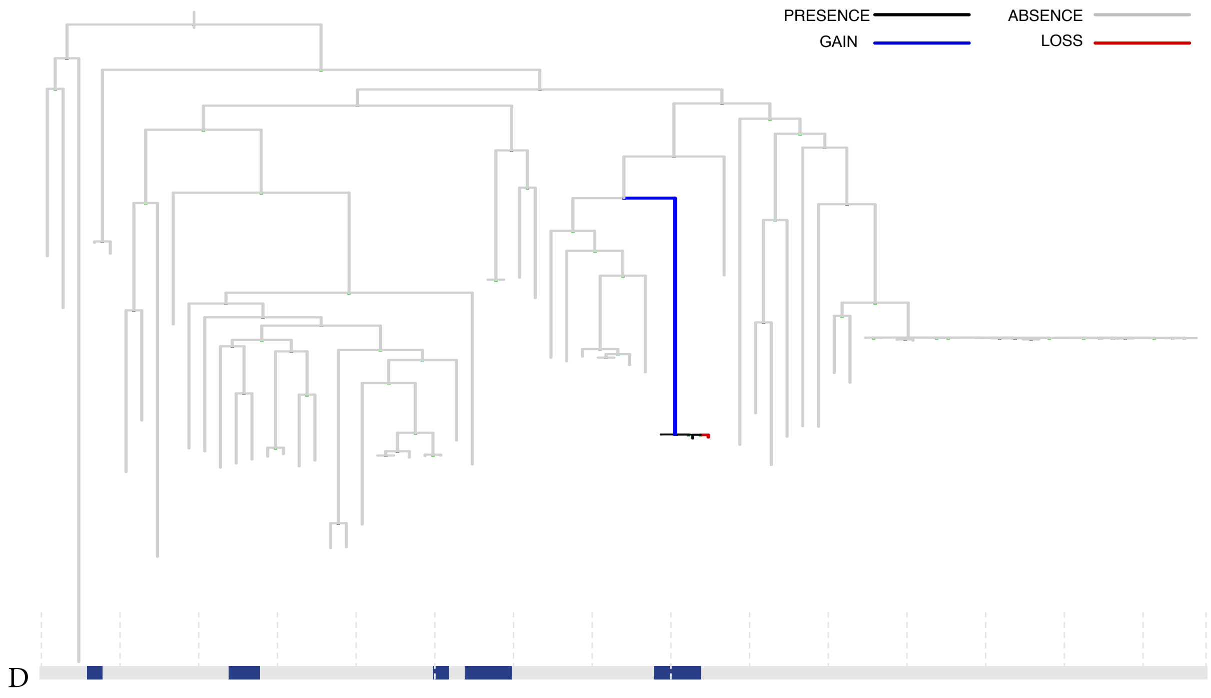

Figure S5. A hierarchical cluster that is split into singletons by CLIME. (a) Patterns of presence and absence of four phylogenetic profiles across the 74 genomes in the phylogenetic tree. (b-e) Mapping of each gene to the reference tree by CLIME. In (b-e) figures, the tree is the phylogenetic tree of 74 genomes; the blue and red lines represent gene gain and loss respectively; In the profiles, the dark blocks represent the presence and the gray means absence.

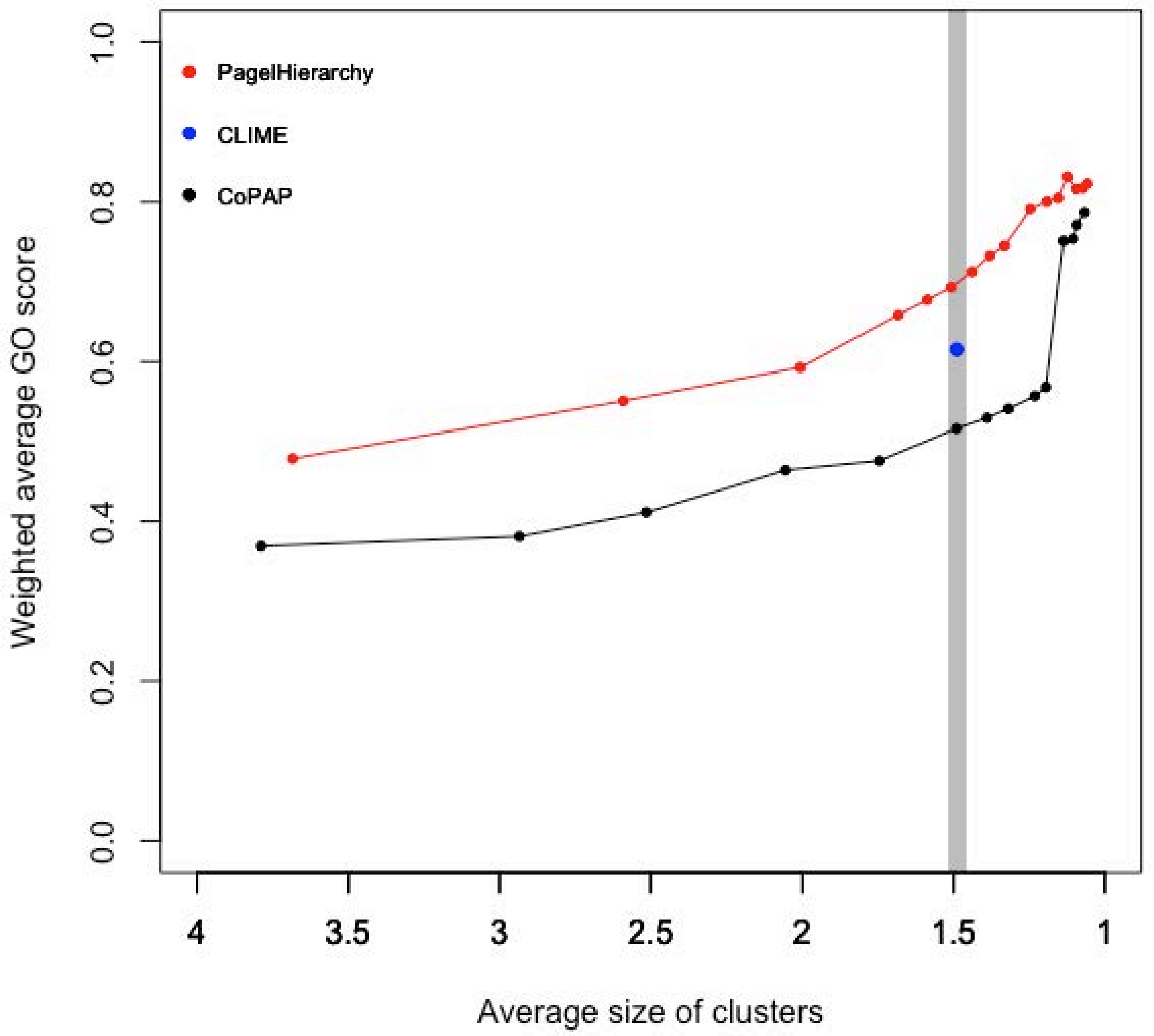

Figure S6. The performance of our method and CoPAP at different cut-offs. The x-axis represents the average size of cluters generated by the corresponding cut-offs and the y-axis is the weighted average GO score. The red, blue and black dots represent our method, CLIME and CoPAP respectively. The three data points in the shaded area are further studied for the significance test in Figure S7.

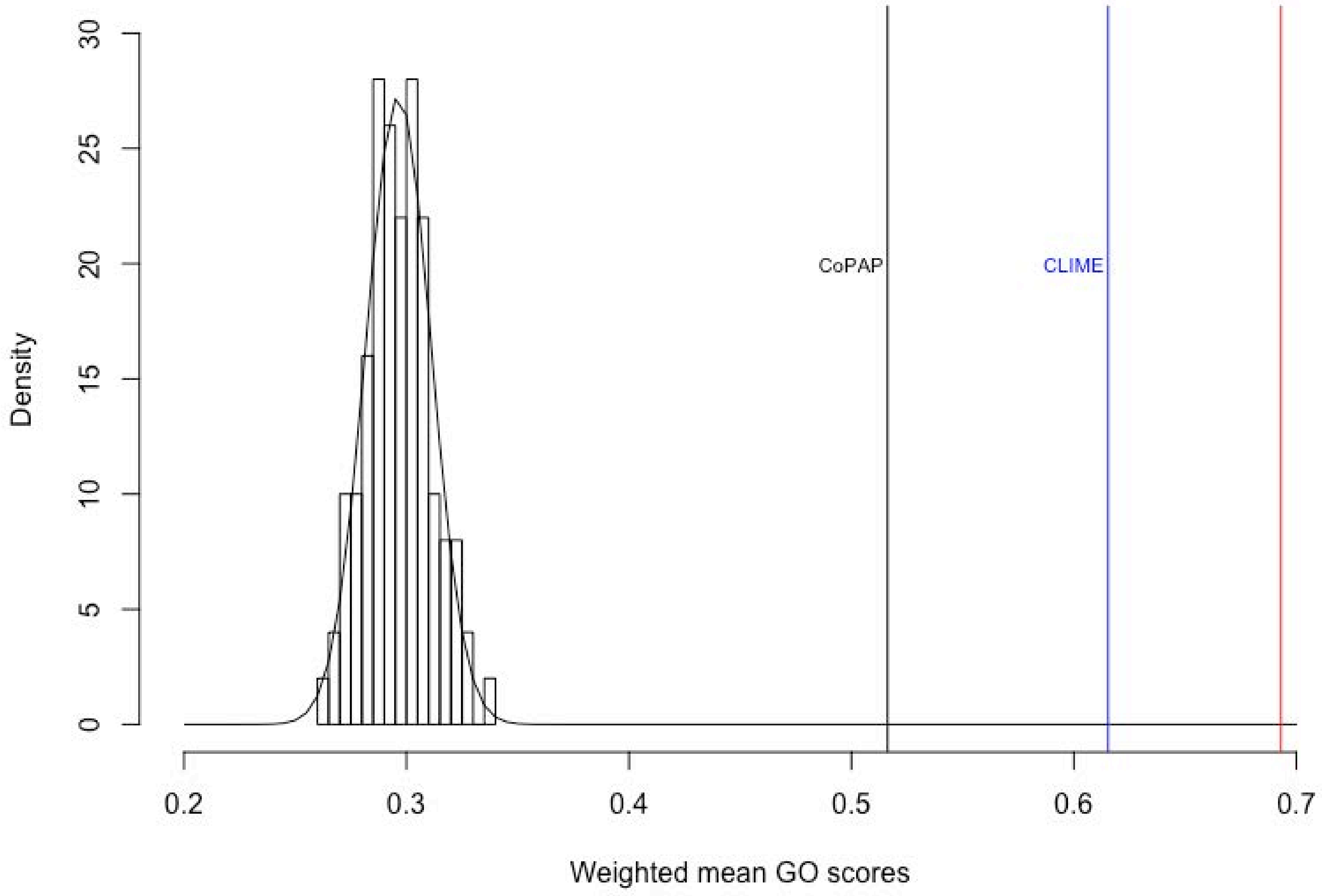

Figure S7. The significance of the performance of three clustering methods. The histogram is generated by 100 randomized assignments of genes corresponding to the size disbribution of CLIME's clustering results. The three vertical lines represent CoPAP, CLIME and our method at the cut-offs in the shaded area of Figure S6.

(a)

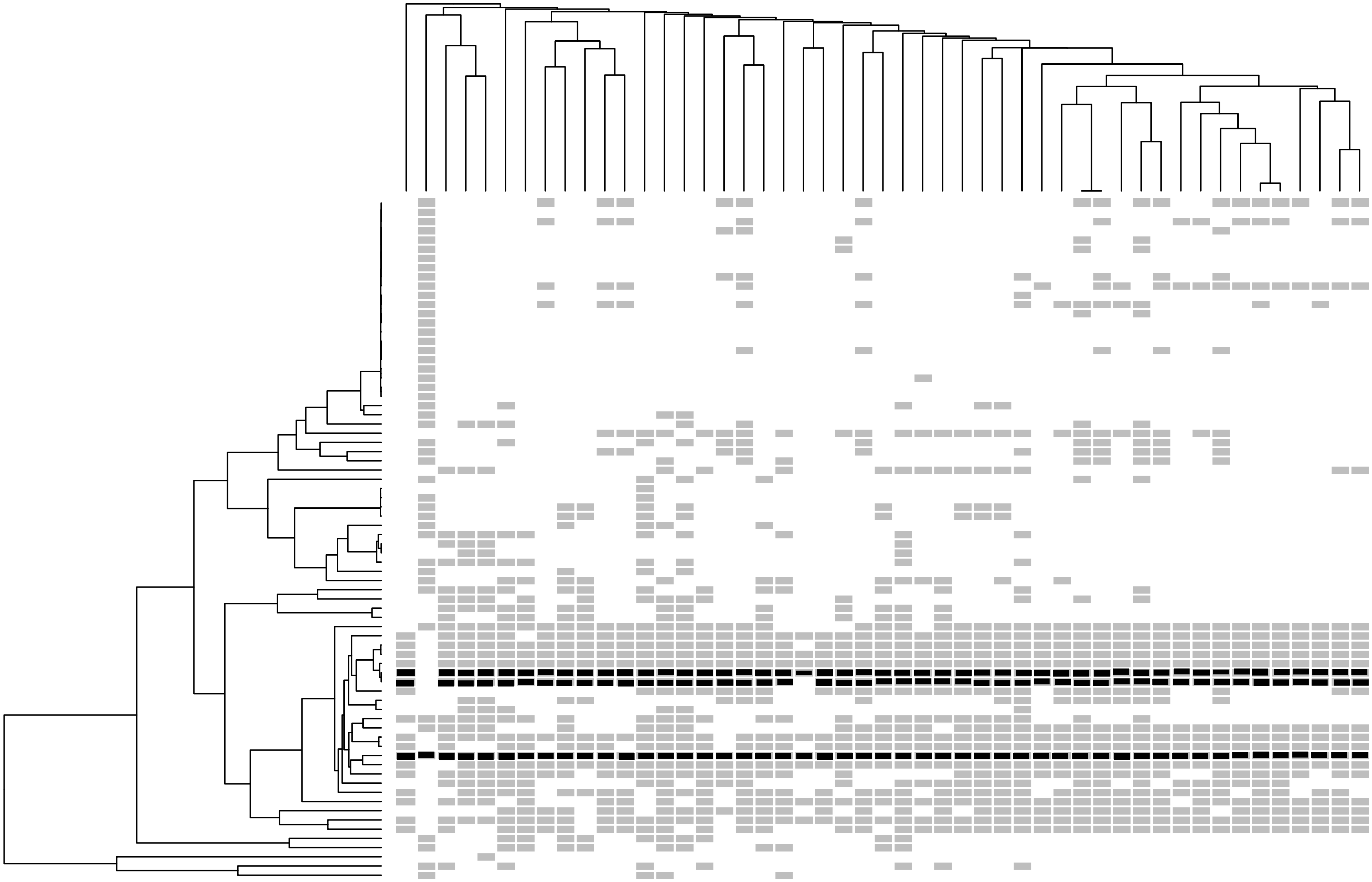

(b)

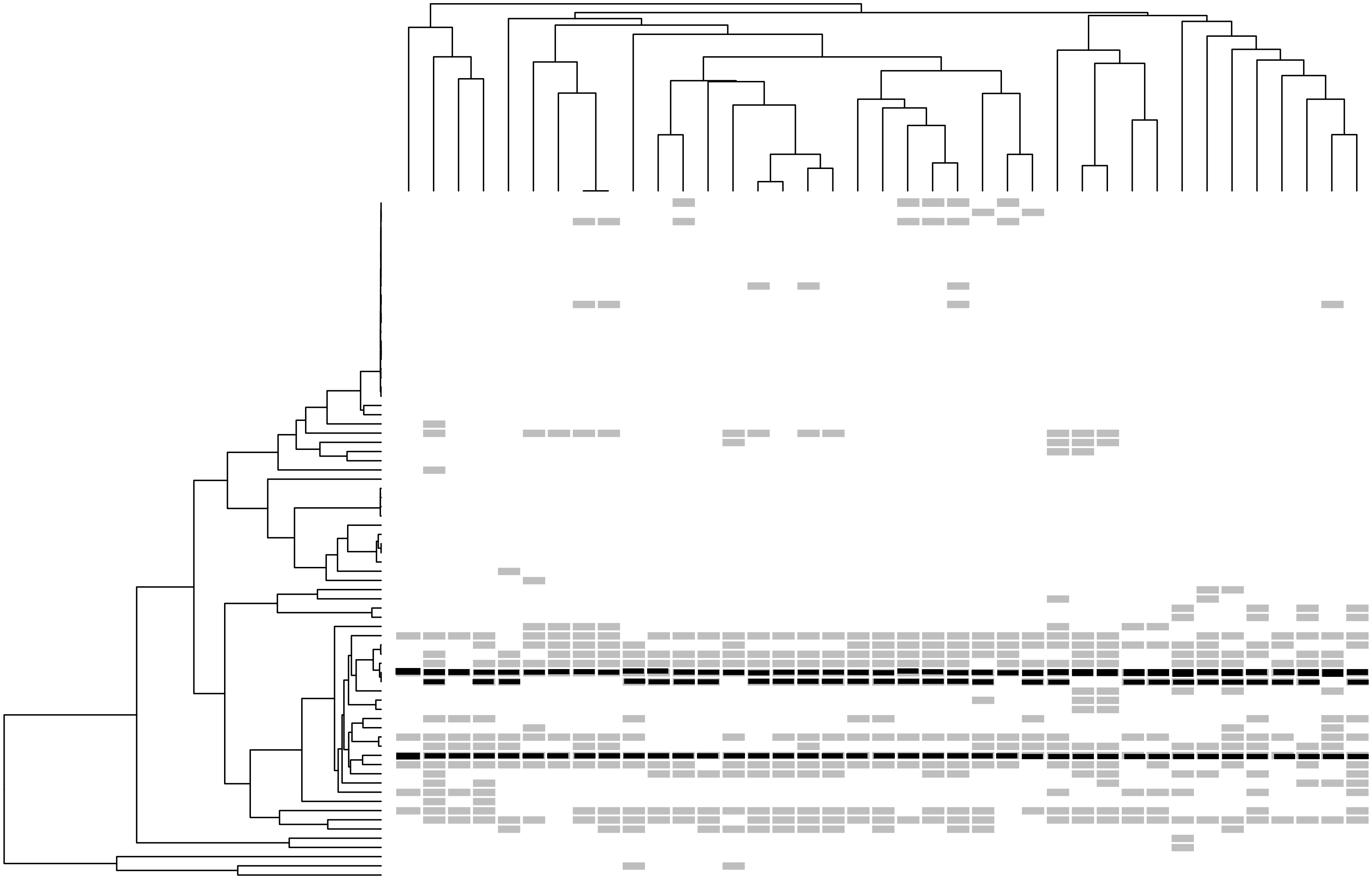

(c)

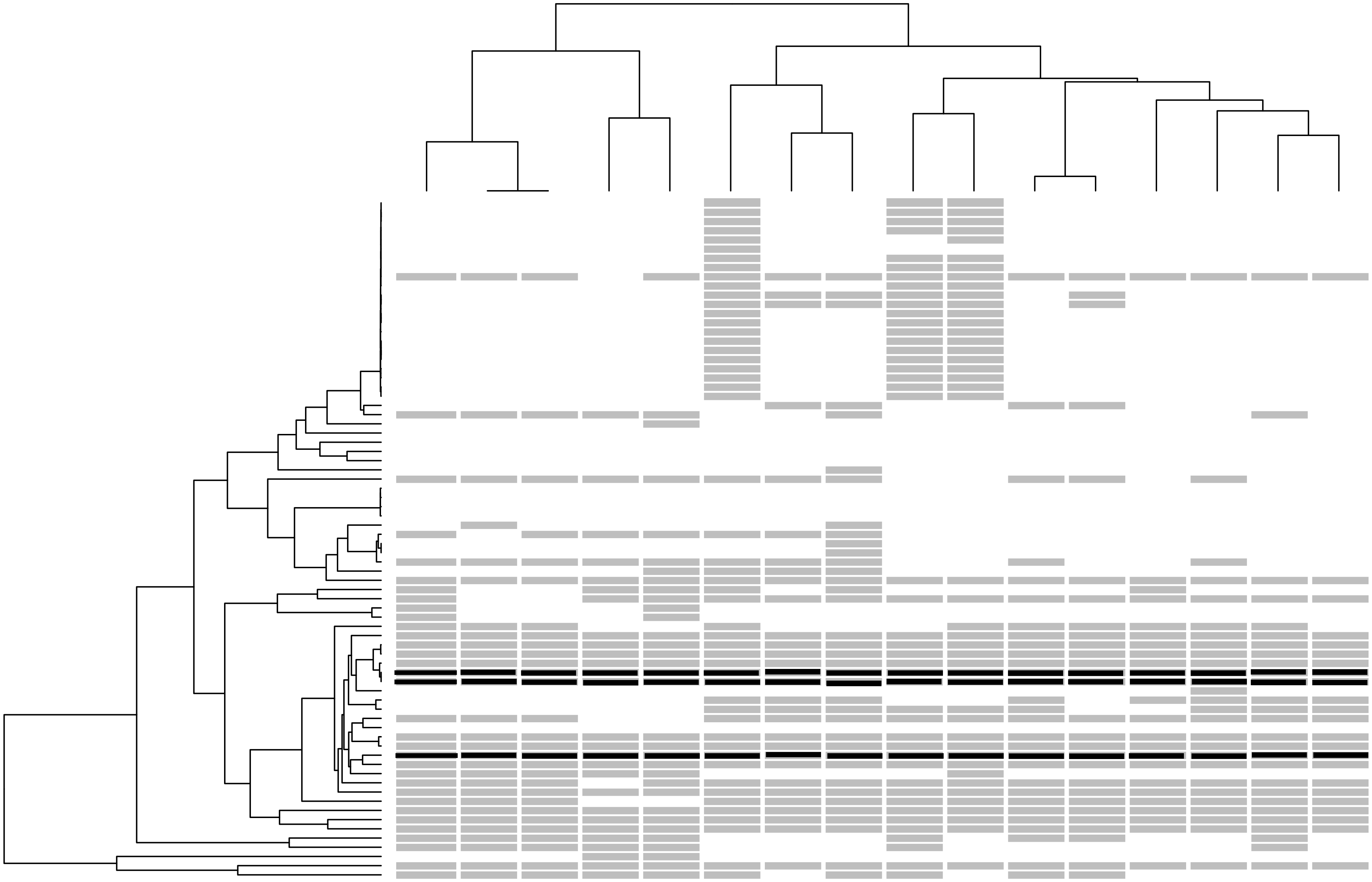

Figure S8. Structure, phylogenetic distribution and functional categories of three clusters with significant over representation of the identified proteins to the two strains of C. bolteae. The three rows of black bars represent C. bolteae 90B7, C. bolteae 90B8 and LachnoZilla from top to bottom.

(a)

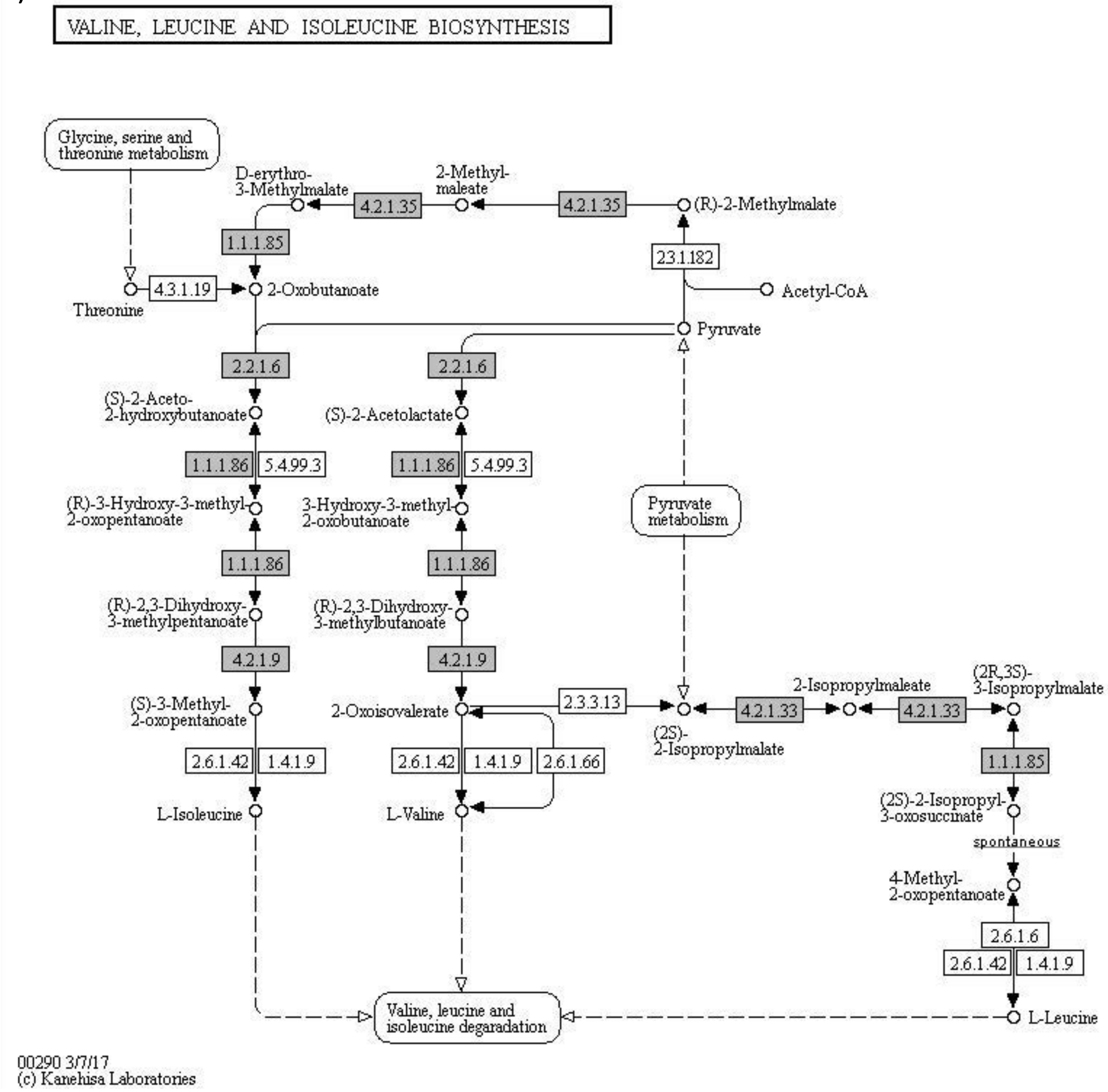
VALINE, LEUCINE AND ISOLEUCINE BIOSYNTHESIS
Glycine, serine and threonine metabolism
D-erythro-3-Methylmalate
2-Methyl-maleate
4.2.1.35
4.2.1.35
(R)-2-Methylmalate
1.1.1.85
2.3.1.182
Threonine
4.3.1.19
2-Oxobutanoate
Acetyl-CoA
Pyruvate
2.2.1.6
2.2.1.6
(S)-2-Aceto-2-hydroxybutanoate
(S)-2-Acetolactate
1.1.1.86
5.4.99.3
1.1.1.86
5.4.99.3
(R)-3-Hydroxy-3-methyl-2-oxopentanoate
3-Hydroxy-3-methyl-2-oxobutanoate
Pyruvate metabolism
1.1.1.86
1.1.1.86
(R)-2,3-Dihydroxy-3-methylpentanoate
(R)-2,3-Dihydroxy-3-methylbutanoate
4.2.1.9
4.2.1.9
(S)-3-Methyl-2-oxopentanoate
2-Oxoisovalerate
2.3.3.13
(2S)-2-Isopropylmalate
4.2.1.33
2-Isopropylmaleate
4.2.1.33
(2R,3S)-3-Isopropylmalate
2.6.1.42
1.4.1.9
2.6.1.42
1.4.1.9
2.6.1.66
1.1.1.85
L-Isoleucine
L-Valine
(2S)-2-Isopropyl-3-oxosuccinate
spontaneous
4-Methyl-2-oxopentanoate
2.6.1.6
2.6.1.42
1.4.1.9
Valine, leucine and isoleucine degaradation
L-Leucine
00290 3/7/17
(c) Kanehisa Laboratories

(b)

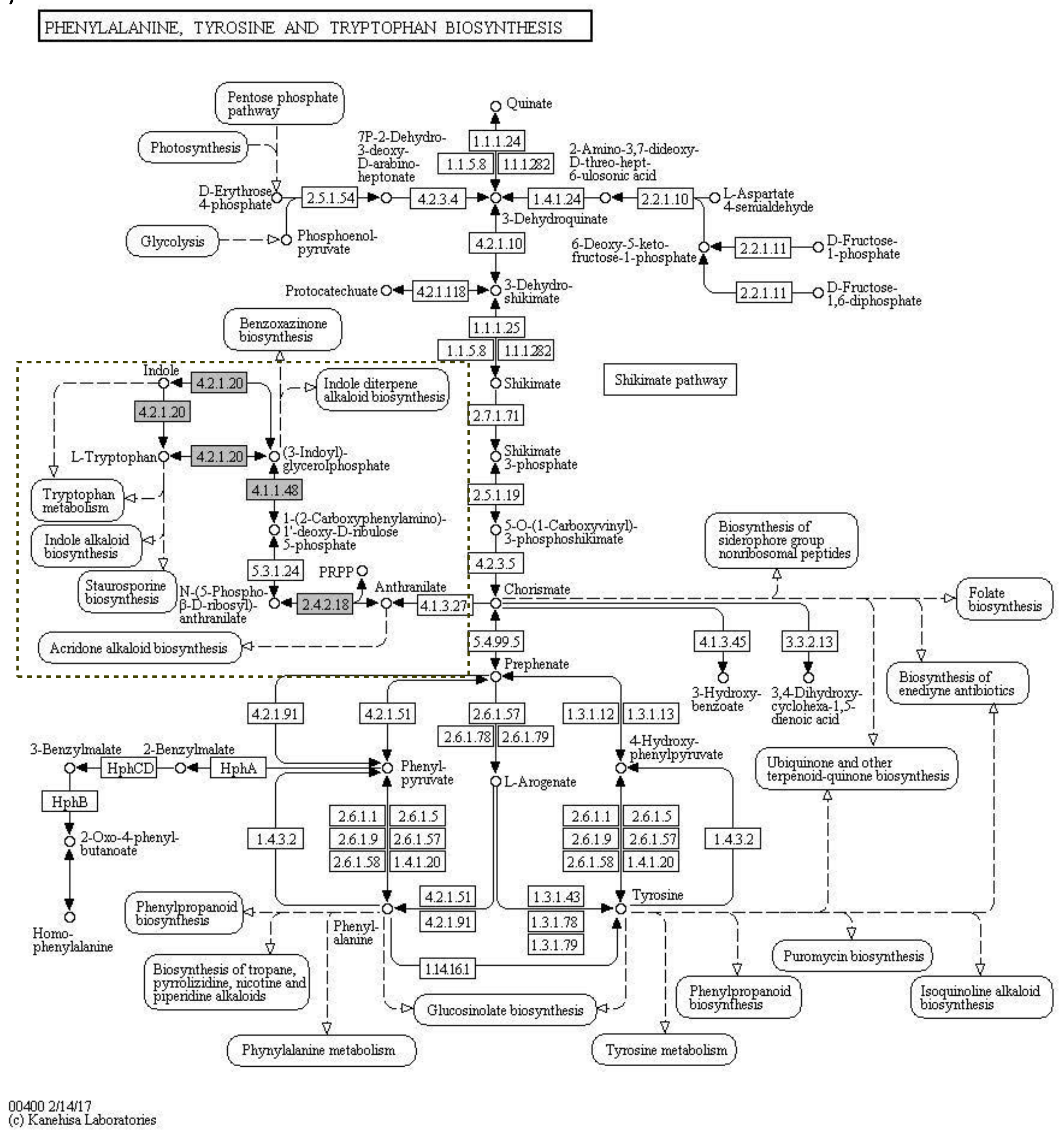

Figure S9. Amino-acid biosynthesis pathway map, indicating steps that are represented in the cluster shown in Figure 5.
