# Supplementary material for: Phylogenetic Clustering of Genes Reveals Shared Evolutionary Trajectories and Putative Gene Functions
Source: Genome Biol Evol. 2018 Aug 20;10(9):2255–65. doi: 10.1093/gbe/evy178 (PMC6130602; doi:10.1093/gbe/evy178)
Supplement: Supplementary Data [file evy178_supp.zip › Tables_Supplementary.pdf]

Table S1. Genomes included in this study. The 73 genomes (in addition to LachnoZilla) have value 1 in the ''Subsampled tree'' column. The SILVA taxonomy for organisms of particular importance that lack official taxonomic IDs are shown.

Class	Genome Name / ID	Subsampled tree	Silva Taxonomy
Clostridia	Lachnospiraceae bacterium 3 1 57FAA CT1	1	
Clostridia	Bacteroides capillosus ATCC 29799 (uid18173)	1	
Clostridia	Bacteroides pectinophilus ATCC 43243 (uid27825)	1	
Clostridia	Caldicellulosiruptor kronotskyensis 2002 (uid52409)	1	
Clostridia	Candidatus Arthromitus SFB 3 (uid69901)	1	
Clostridia	Candidatus Arthromitus SFB 4 (uid69905)	1	
Clostridia	Candidatus Arthromitus SFB mouse NYU (uid71495)	1	
Clostridia	Candidatus Arthromitus SFB mouse SU (uid76365)	1	
Clostridia	Catonella morbi ATCC 51271 (uid33121)	1	
Clostridia	Clostridiales bacterium 1 7 47FAA (uid30067)	1	Clostridiales;Lachnospiraceae;Lachnoclostridium
Clostridia	Clostridiales bacterium VE202-28)	1	No Silva taxonomy
Clostridia	Clostridium 7 3 54FAA (uid40017)	1	Clostridiales;Lachnospiraceae;Lachnoclostridium
Clostridia	Clostridium bartlettii DORA 8 9 (uid221485)	1	
Clostridia	Clostridium BNL1100 (uid81613)	1	Clostridiales;Ruminococcaceae;Ruminiclostridium
Clostridia	Clostridium bolteae 90B7 (uid64851)	1	
Clostridia	Clostridium bolteae 90B8 (uid64853)	1	
Clostridia	Clostridium botulinum C Eklund (uid20017)	1	
Clostridia	Clostridium botulinum CFSAN002367 (uid192664)	1	
Clostridia	Clostridium botulinum CFSAN002369 (uid192666)	1	
Clostridia	Clostridium botulinum F Langeland (uid19519)	1	
Clostridia	Clostridium clostridioforme 90A4 (uid64861)	1	
Clostridia	Clostridium difficile 6042 (uid85803)	1	
Clostridia	Clostridium difficile CD196 (uid38037)	1	
Clostridia	Clostridium difficile CD200 (uid85775)	1	
Clostridia	Clostridium difficile CD92 (uid85731)	1	
Clostridia	Clostridium difficile DA00062 (uid85811)	1	
Clostridia	Clostridium difficile DA00114 (uid85815)	1	
Clostridia	Clostridium difficile E1 (uid238025)	1	
Clostridia	Clostridium difficile E28 (uid222738)	1	
Clostridia	Clostridium difficile F665 (uid175462)	1	
Clostridia	Clostridium difficile NAP07 (uid31439)	1	
Clostridia	Clostridium difficile P23 (uid85989)	1	
Clostridia	Clostridium difficile P50 (uid86025)	1	
Clostridia	Clostridium difficile P53 (uid86031)	1	
Clostridia	Clostridium difficile P64 (uid86039)	1	
Clostridia	Clostridium difficile P70 (uid86045)	1	
Clostridia	Clostridium difficile P73 (uid86051)	1	
Clostridia	Clostridium difficile T22 (uid222725)	1	
Clostridia	Clostridium difficile T3 (uid222752)	1	
Clostridia	Clostridium difficile Y10 (uid85921)	1	
Clostridia	Clostridium difficile Y270 (uid85945)	1	
Clostridia	Clostridium hylemonae DSM 15053 (uid30369)	1	
Clostridia	Clostridium L2 50 (uid18211)	1	Clostridiales;Lachnospiraceae;Dorea
Clostridia	Clostridium Maddingley MBC34 26 (uid167830)	1	
Clostridia	Clostridium sordellii ATCC 9714 (uid188695)	1	
Clostridia	Clostridium sp. ASF356)	1	Clostridiales;Lachnospiraceae;uncultured
Clostridia	Clostridium sporogenes ATCC 15579 (uid20549)	1	
Clostridia	Clostridium stercorarium DSM 8532 (uid180992)	1	
Clostridia	Clostridium thermocellum ATCC 27405 (uid314)	1	
Clostridia	Desulfitobacterium hafniense DP7 (uid30023)	1	
Clostridia	Desulfotomaculum gibsoniae DSM 7213 (uid59873)	1	
Clostridia	Dorea longicatena DSM 13814 (uid18157)	1	
Clostridia	Eubacteriaceae bacterium CM2 (uid49889)	1	Peptoanaerobacter stomatis
Clostridia	Eubacterium nodatum ATCC 33099 (uid89653)	1	
Clostridia	Eubacterium rectale (uid39159)	1	
Clostridia	Finegoldia magna SY403409CC001050417 (uid51081)	1	
Clostridia	Lachnospiraceae bacterium 2 1 46FAA (uid46369)	1	Clostridiales;Lachnospiraceae;Lachnoclostridium
Clostridia	Lachnospiraceae bacterium A4 (uid175979)	1	Clostridiales;Lachnospiraceae;Lachnospiraceae NK4A136 group
Clostridia	Lachnospiraceae bacterium ICM7 (uid78559)	1	Clostridiales;Lachnospiraceae;Lachnoanaerobaculum
Clostridia	Moorella thermoacetica Y72 (uid238520)	1	
Clostridia	Oribacterium ACB7 (uid49879)	1	
Clostridia	Oribacterium sinus F0268 (uid34635)	1	
Clostridia	Oscillibacter valericigenes (uid41823)	1	
Clostridia	Peptoniphilus duerdenii ATCC BAA 1640 (uid50529)	1	
Clostridia	Peptoniphilus oral taxon 386 F0131 (uid41969)	1	
Clostridia	Pseudoramibacter alactolyticus ATCC 23263 (uid52995)	1	
Clostridia	Ruminococcus obeum (uid39167)	1	
Clostridia	Ruminococcus obeum ATCC 29174 (uid18147)	1	
Clostridia	Thermaerobacter marianensis DSM 12885 (uid38025)	1	
Clostridia	Thermincola JR (uid41467)	1	
Clostridia	Thermoanaerobacter wiegelii Rt8 B1 (uid42251)	1	
Clostridia	Thermoanaerobacter X514 (uid16394)	1	

Clostridia	Thermobrachium celere DSM 8682 (uid61471)	1	
Clostridia	Thermodesulfobium narugense DSM 14796 (uid46673)	1	
Clostridia	Acetobacterium woodii DSM 1030 (uid60713)	0	
Clostridia	Acetohalobium arabaticum DSM 5501 (uid32769)	0	
Clostridia	Alkaliphilus metalliredigens QYMF (uid13006)	0	
Clostridia	Alkaliphilus oremlandii OhILAs (uid16083)	0	
Clostridia	Ammonifex degensii KC4 (uid12390)	0	
Clostridia	Anaerocellum thermophilum DSM 6725 (uid29407)	0	
Clostridia	Anaerococcus hydrogenalis ACS 025 V Sch4 (uid52027)	0	
Clostridia	Anaerococcus hydrogenalis DSM 7454 (uid30747)	0	
Clostridia	Anaerococcus lactolyticus ATCC 51172 (uid30951)	0	
Clostridia	Anaerococcus prevotii ACS 065 V Col13 (uid52025)	0	
Clostridia	Anaerococcus prevotii DSM 20548 (uid29533)	0	
Clostridia	Anaerococcus tetradius ATCC 35098 (uid31433)	0	
Clostridia	Anaerococcus vaginalis ATCC 51170 (uid38727)	0	
Clostridia	Anaerofustis stercorihominis DSM 17244 (uid19657)	0	
Clostridia	Anaerostipes 3 2 56FAA (uid39999)	0	
Clostridia	Anaerostipes caccae DSM 14662 (uid18213)	0	
Clostridia	Anaerotruncus colihominis DSM 17241 (uid19659)	0	
Clostridia	Anaerotruncus G3 2012 (uid175989)	0	
Bacilli	Bacillus subtilis subsp. subtilis str. 168)	0	
Clostridia	Blautia hansenii DSM 20583 (uid30021)	0	
Clostridia	Blautia hydrogenotrophica DSM 10507 (uid21007)	0	
Clostridia	Blautia KLE 1732 (uid173639)	0	
Clostridia	Blautia sp. KLE 1732)	0	
Clostridia	Bryantella formatexigens DSM 14469 (uid21051)	0	
Clostridia	butyrate producing bacterium SM4 1 (uid45955)	0	
Clostridia	butyrate producing bacterium SS3 4 (uid39155)	0	
Clostridia	butyrate producing bacterium SSC 2 (uid45957)	0	
Clostridia	Butyricicoccus pullicaecorum 1 2 (uid170345)	0	
Clostridia	Butyrivibrio crossotus DSM 2876 (uid28999)	0	
Clostridia	Butyrivibrio fibrisolvens (uid39147)	0	
Clostridia	Butyrivibrio proteoclasticus B316 (uid29153)	0	
Clostridia	Butyrivibrio proteoclasticus B316)	0	
Clostridia	Caldanaerobacter subterraneus yonseiensis KB 1 (uid215704)	0	
Clostridia	Caldicellulosiruptor hydrothermalis 108 (uid40831)	0	
Clostridia	Caldicellulosiruptor kristjanssonii 177R1B (uid41727)	0	
Clostridia	Caldicellulosiruptor lactoaceticus 6A (uid40219)	0	
Clostridia	Caldicellulosiruptor obsidiansis OB47 (uid40355)	0	
Clostridia	Caldicellulosiruptor owensensis OL (uid40833)	0	
Clostridia	Caldicellulosiruptor saccharolyticus DSM 8903 (uid13466)	0	
Clostridia	Caloramator australicus RC3 (uid49895)	0	
Clostridia	Candidatus Arthromitus SFB 1 (uid68497)	0	
Clostridia	Candidatus Arthromitus SFB 2 (uid68847)	0	
Clostridia	Candidatus Arthromitus SFB 5 (uid69909)	0	
Clostridia	Candidatus Arthromitus SFB co (uid69911)	0	
Clostridia	Candidatus Arthromitus SFB mouse Japan (uid66727)	0	
Clostridia	Candidatus Arthromitus SFB mouse Yit (uid67835)	0	
Clostridia	Candidatus Arthromitus SFB rat Yit (uid67837)	0	
Clostridia	Candidatus Desulforudis audaxviator MP104C (uid21047)	0	
Clostridia	Carboxydibrachium pacificum DSM 12653 (uid19289)	0	
Clostridia	Carboxydothermus hydrogenoformans Z-2901 (uid253)	0	
Clostridia	Clostridiaceae bacterium L21 TH D2 (uid196482)	0	
Clostridia	Clostridiales bacterium BV3Ac2 (uid89635)	0	
Clostridia	Clostridiales bacterium BV3C26 (uid89637)	0	
Clostridia	Clostridiales bacterium NK3B98)	0	
Clostridia	Clostridiales bacterium OBRC5 5 (uid78963)	0	
Clostridia	Clostridiales bacterium VE202-03)	0	
Clostridia	Clostridiales bacterium VE202-07)	0	
Clostridia	Clostridiales bacterium VE202-09)	0	
Clostridia	Clostridiales bacterium VE202-13)	0	
Clostridia	Clostridiales bacterium VE202-14)	0	
Clostridia	Clostridiales bacterium VE202-15)	0	
Clostridia	Clostridiales bacterium VE202-16)	0	
Clostridia	Clostridiales bacterium VE202-21)	0	
Clostridia	Clostridiales bacterium VE202-26)	0	
Clostridia	Clostridiales bacterium VE202-27)	0	Clostridiales;Lachnospiraceae;Eisenbergiella
Clostridia	Clostridiales bacterium VE202-29)	0	Clostridiales;Lachnospiraceae;Eisenbergiella
Clostridia	Clostridiales genomosp BVAB3 UPII9 5 (uid42555)	0	
Clostridia	Clostridium 7 2 43FAA (uid32457)	0	
Clostridia	Clostridium acetobutylicum (uid77)	0	
Clostridia	Clostridium acetobutylicum DSM 1731 (uid63241)	0	
Clostridia	Clostridium acetobutylicum EA 2018 (uid50455)	0	
Clostridia	Clostridium acidurici 9a (uid79221)	0	
Clostridia	Clostridium ASBs410 (uid195881)	0	
Clostridia	Clostridium ASF356 (uid175999)	0	

Clostridia	Clostridium ASF502 (uid176003)	0
Clostridia	Clostridium asparagiforme DSM 15981 (uid29263)	0
Clostridia	Clostridium autoethanogenum DSM 10061 (uid219420)	0
Clostridia	Clostridium bartlettii DSM 16795 (uid19661)	0
Clostridia	Clostridium beijerinckii NCIMB 8052 (uid12637)	0
Clostridia	Clostridium bifermentans ATCC 19299 (uid175455)	0
Clostridia	Clostridium bifermentans ATCC 638 (uid175456)	0
Clostridia	Clostridium BL8 (uid210530)	0
Clostridia	Clostridium bolteae 90A5 (uid64845)	0
Clostridia	Clostridium bolteae 90A5)	0
Clostridia	Clostridium bolteae 90A9 (uid64847)	0
Clostridia	Clostridium bolteae 90A9)	0
Clostridia	Clostridium bolteae 90B3 (uid64849)	0
Clostridia	Clostridium bolteae 90B3)	0
Clostridia	Clostridium bolteae 90B7)	0
Clostridia	Clostridium bolteae 90B8)	0
Clostridia	Clostridium bolteae ATCC BAA 613 (uid18165)	0
Clostridia	Clostridium bolteae ATCC BAA-613)	0
Clostridia	Clostridium botulinum 84 (uid178523)	0
Clostridia	Clostridium botulinum A (uid193)	0
Clostridia	Clostridium botulinum A ATCC 19397 (uid19517)	0
Clostridia	Clostridium botulinum A Hall (uid19521)	0
Clostridia	Clostridium botulinum A1 CFSAN002368 (uid192665)	0
Clostridia	Clostridium botulinum A2 Kyoto (uid29859)	0
Clostridia	Clostridium botulinum A3 Loch Maree (uid28507)	0
Clostridia	Clostridium botulinum B Eklund 17B (uid28857)	0
Clostridia	Clostridium botulinum B Osaka05 (uid227279)	0
Clostridia	Clostridium botulinum B1 Okra (uid28505)	0
Clostridia	Clostridium botulinum Ba4 657 (uid29077)	0
Clostridia	Clostridium botulinum Bf (uid20015)	0
Clostridia	Clostridium botulinum BKT015925 (uid60407)	0
Clostridia	Clostridium botulinum C Stockholm (uid60409)	0
Clostridia	Clostridium botulinum CFSAN001627 (uid175310)	0
Clostridia	Clostridium botulinum CFSAN001628 (uid175309)	0
Clostridia	Clostridium botulinum D 1873 (uid33173)	0
Clostridia	Clostridium botulinum E1 BoNT E Beluga (uid29861)	0
Clostridia	Clostridium botulinum E3 Alaska E43 (uid28855)	0
Clostridia	Clostridium botulinum F 230613 (uid47575)	0
Clostridia	Clostridium botulinum H04402 065 (uid61511)	0
Clostridia	Clostridium botulinum NCTC 2916 (uid20013)	0
Clostridia	Clostridium butyricum 5521 (uid20023)	0
Clostridia	Clostridium butyricum 60E 3 (uid64855)	0
Clostridia	Clostridium butyricum DKU 01 (uid193356)	0
Clostridia	Clostridium butyricum DORA 1 (uid221486)	0
Clostridia	Clostridium butyricum E4 BoNT E BL5262 (uid34907)	0
Clostridia	Clostridium carboxidivorans P7 (uid29495)	0
Clostridia	Clostridium carboxidivorans P7 (uid33115)	0
Clostridia	Clostridium celatum DSM 1785 (uid30375)	0
Clostridia	Clostridium cellulolyticum H10 (uid17419)	0
Clostridia	Clostridium cellulovorans 743B (uid32609)	0
Clostridia	Clostridium cf saccharolyticum K10 (uid45855)	0
Clostridia	Clostridium chauvoei JF4335 (uid211323)	0
Clostridia	Clostridium citroniae WAL 17108 (uid46385)	0
Clostridia	Clostridium clariflavum DSM 19732 (uid72805)	0
Clostridia	Clostridium clostridioforme 2 1 49FAA (uid46389)	0
Clostridia	Clostridium clostridioforme 2 1 49FAA)	0
Clostridia	Clostridium clostridioforme 90A1 (uid64857)	0
Clostridia	Clostridium clostridioforme 90A1)	0
Clostridia	Clostridium clostridioforme 90A3 (uid64859)	0
Clostridia	Clostridium clostridioforme 90A3)	0
Clostridia	Clostridium clostridioforme 90A4)	0
Clostridia	Clostridium clostridioforme 90A6 (uid64863)	0
Clostridia	Clostridium clostridioforme 90A6)	0
Clostridia	Clostridium clostridioforme 90A7 (uid64865)	0
Clostridia	Clostridium clostridioforme 90A7)	0
Clostridia	Clostridium clostridioforme 90A8 (uid64867)	0
Clostridia	Clostridium clostridioforme 90A8)	0
Clostridia	Clostridium clostridioforme 90B1 (uid64869)	0
Clostridia	Clostridium clostridioforme 90B1)	0
Clostridia	Clostridium clostridioforme AGR2157)	0
Clostridia	Clostridium clostridioforme CM201 (uid64871)	0
Clostridia	Clostridium clostridioforme CM201)	0
Clostridia	Clostridium colicanis 209318 (uid64873)	0
Clostridia	Clostridium D5 (uid32459)	0
Clostridia	Clostridium difficile (uid238404)	0
Clostridia	Clostridium difficile 002 P50 2011 (uid64719)	0

Clostridia	Clostridium difficile 050 P50 2011 (uid64721)	0
Clostridia	Clostridium difficile 342 (uid85787)	0
Clostridia	Clostridium difficile 6041 (uid85801)	0
Clostridia	Clostridium difficile 6057 (uid85807)	0
Clostridia	Clostridium difficile 630 (uid78)	0
Clostridia	Clostridium difficile 655 (uid85791)	0
Clostridia	Clostridium difficile 70 100 2010 (uid65135)	0
Clostridia	Clostridium difficile 824 (uid85793)	0
Clostridia	Clostridium difficile 840 (uid85795)	0
Clostridia	Clostridium difficile 842 (uid85799)	0
Clostridia	Clostridium difficile CD002 (uid222724)	0
Clostridia	Clostridium difficile CD104 (uid85733)	0
Clostridia	Clostridium difficile CD109 (uid85735)	0
Clostridia	Clostridium difficile CD111 (uid85737)	0
Clostridia	Clostridium difficile CD113 (uid85739)	0
Clostridia	Clostridium difficile CD127 (uid85741)	0
Clostridia	Clostridium difficile CD129 (uid85743)	0
Clostridia	Clostridium difficile CD13 (uid85683)	0
Clostridia	Clostridium difficile CD131 (uid85745)	0
Clostridia	Clostridium difficile CD132 (uid85747)	0
Clostridia	Clostridium difficile CD133 (uid85749)	0
Clostridia	Clostridium difficile CD144 (uid85751)	0
Clostridia	Clostridium difficile CD149 (uid85753)	0
Clostridia	Clostridium difficile CD159 (uid85755)	0
Clostridia	Clostridium difficile CD160 (uid85757)	0
Clostridia	Clostridium difficile CD165 (uid85759)	0
Clostridia	Clostridium difficile CD166 (uid85761)	0
Clostridia	Clostridium difficile CD169 (uid85763)	0
Clostridia	Clostridium difficile CD17 (uid85685)	0
Clostridia	Clostridium difficile CD170 (uid85765)	0
Clostridia	Clostridium difficile CD175 (uid85767)	0
Clostridia	Clostridium difficile CD178 (uid85769)	0
Clostridia	Clostridium difficile CD18 (uid85687)	0
Clostridia	Clostridium difficile CD181 (uid85771)	0
Clostridia	Clostridium difficile CD196 (uid85773)	0
Clostridia	Clostridium difficile CD201 (uid85777)	0
Clostridia	Clostridium difficile CD206 (uid85781)	0
Clostridia	Clostridium difficile CD21 (uid85689)	0
Clostridia	Clostridium difficile CD211 (uid85783)	0
Clostridia	Clostridium difficile CD212 (uid85785)	0
Clostridia	Clostridium difficile CD22 (uid85691)	0
Clostridia	Clostridium difficile CD3 (uid85677)	0
Clostridia	Clostridium difficile CD34 (uid85693)	0
Clostridia	Clostridium difficile CD38 (uid85695)	0
Clostridia	Clostridium difficile CD39 (uid85697)	0
Clostridia	Clostridium difficile CD40 (uid85699)	0
Clostridia	Clostridium difficile CD41 (uid85701)	0
Clostridia	Clostridium difficile CD42 (uid85703)	0
Clostridia	Clostridium difficile CD43 (uid85705)	0
Clostridia	Clostridium difficile CD44 (uid85707)	0
Clostridia	Clostridium difficile CD45 (uid85709)	0
Clostridia	Clostridium difficile CD46 (uid85711)	0
Clostridia	Clostridium difficile CD47 (uid85713)	0
Clostridia	Clostridium difficile CD49 (uid85715)	0
Clostridia	Clostridium difficile CD51 (uid85717)	0
Clostridia	Clostridium difficile CD68 (uid85719)	0
Clostridia	Clostridium difficile CD69 (uid85721)	0
Clostridia	Clostridium difficile CD70 (uid85723)	0
Clostridia	Clostridium difficile CD8 (uid85679)	0
Clostridia	Clostridium difficile CD86 (uid85725)	0
Clostridia	Clostridium difficile CD88 (uid85727)	0
Clostridia	Clostridium difficile CD9 (uid85681)	0
Clostridia	Clostridium difficile CD90 (uid85729)	0
Clostridia	Clostridium difficile DA00044 (uid85809)	0
Clostridia	Clostridium difficile DA00065 (uid85813)	0
Clostridia	Clostridium difficile DA00126 (uid85817)	0
Clostridia	Clostridium difficile DA00128 (uid85819)	0
Clostridia	Clostridium difficile DA00129 (uid85821)	0
Clostridia	Clostridium difficile DA00130 (uid85823)	0
Clostridia	Clostridium difficile DA00131 (uid85825)	0
Clostridia	Clostridium difficile DA00132 (uid85827)	0
Clostridia	Clostridium difficile DA00134 (uid85829)	0
Clostridia	Clostridium difficile DA00141 (uid85831)	0
Clostridia	Clostridium difficile DA00142 (uid85833)	0
Clostridia	Clostridium difficile DA00145 (uid85835)	0
Clostridia	Clostridium difficile DA00149 (uid85837)	0

Clostridia	Clostridium difficile DA00154 (uid85839)	0
Clostridia	Clostridium difficile DA00160 (uid85841)	0
Clostridia	Clostridium difficile DA00165 (uid85843)	0
Clostridia	Clostridium difficile DA00167 (uid85845)	0
Clostridia	Clostridium difficile DA00174 (uid85847)	0
Clostridia	Clostridium difficile DA00183 (uid85849)	0
Clostridia	Clostridium difficile DA00189 (uid85851)	0
Clostridia	Clostridium difficile DA00191 (uid85853)	0
Clostridia	Clostridium difficile DA00193 (uid85855)	0
Clostridia	Clostridium difficile DA00195 (uid85857)	0
Clostridia	Clostridium difficile DA00196 (uid85859)	0
Clostridia	Clostridium difficile DA00197 (uid85861)	0
Clostridia	Clostridium difficile DA00203 (uid85863)	0
Clostridia	Clostridium difficile DA00210 (uid85865)	0
Clostridia	Clostridium difficile DA00211 (uid85867)	0
Clostridia	Clostridium difficile DA00212 (uid85869)	0
Clostridia	Clostridium difficile DA00215 (uid85871)	0
Clostridia	Clostridium difficile DA00216 (uid85873)	0
Clostridia	Clostridium difficile DA00232 (uid85875)	0
Clostridia	Clostridium difficile DA00238 (uid85877)	0
Clostridia	Clostridium difficile DA00244 (uid85879)	0
Clostridia	Clostridium difficile DA00245 (uid85881)	0
Clostridia	Clostridium difficile DA00246 (uid85883)	0
Clostridia	Clostridium difficile DA00256 (uid85885)	0
Clostridia	Clostridium difficile DA00261 (uid85887)	0
Clostridia	Clostridium difficile DA00273 (uid85889)	0
Clostridia	Clostridium difficile DA00275 (uid85891)	0
Clostridia	Clostridium difficile DA00305 (uid85893)	0
Clostridia	Clostridium difficile DA00306 (uid85895)	0
Clostridia	Clostridium difficile DA00307 (uid85897)	0
Clostridia	Clostridium difficile DA00310 (uid85899)	0
Clostridia	Clostridium difficile DA00313 (uid85901)	0
Clostridia	Clostridium difficile E10 (uid222733)	0
Clostridia	Clostridium difficile E12 (uid222727)	0
Clostridia	Clostridium difficile E13 (uid222742)	0
Clostridia	Clostridium difficile E14 (uid222726)	0
Clostridia	Clostridium difficile E15 (uid222750)	0
Clostridia	Clostridium difficile E16 (uid222736)	0
Clostridia	Clostridium difficile E19 (uid222729)	0
Clostridia	Clostridium difficile E23 (uid222731)	0
Clostridia	Clostridium difficile E24 (uid222744)	0
Clostridia	Clostridium difficile E25 (uid222734)	0
Clostridia	Clostridium difficile E7 (uid222748)	0
Clostridia	Clostridium difficile E9 (uid222745)	0
Clostridia	Clostridium difficile F152 (uid85903)	0
Clostridia	Clostridium difficile F200 (uid175457)	0
Clostridia	Clostridium difficile F249 (uid85907)	0
Clostridia	Clostridium difficile F253 (uid85909)	0
Clostridia	Clostridium difficile F314 (uid85911)	0
Clostridia	Clostridium difficile F480 (uid175458)	0
Clostridia	Clostridium difficile F501 (uid85919)	0
Clostridia	Clostridium difficile F525 (uid175459)	0
Clostridia	Clostridium difficile F548 (uid175460)	0
Clostridia	Clostridium difficile F601 (uid175461)	0
Clostridia	Clostridium difficile NAP08 (uid31441)	0
Clostridia	Clostridium difficile P1 (uid85961)	0
Clostridia	Clostridium difficile P11 (uid85977)	0
Clostridia	Clostridium difficile P13 (uid85979)	0
Clostridia	Clostridium difficile P15 (uid85981)	0
Clostridia	Clostridium difficile P19 (uid85983)	0
Clostridia	Clostridium difficile P2 (uid85963)	0
Clostridia	Clostridium difficile P20 (uid85985)	0
Clostridia	Clostridium difficile P21 (uid85987)	0
Clostridia	Clostridium difficile P24 (uid85991)	0
Clostridia	Clostridium difficile P25 (uid85993)	0
Clostridia	Clostridium difficile P28 (uid85995)	0
Clostridia	Clostridium difficile P29 (uid85997)	0
Clostridia	Clostridium difficile P3 (uid85965)	0
Clostridia	Clostridium difficile P30 (uid85999)	0
Clostridia	Clostridium difficile P31 (uid86001)	0
Clostridia	Clostridium difficile P32 (uid86003)	0
Clostridia	Clostridium difficile P33 (uid86005)	0
Clostridia	Clostridium difficile P36 (uid86011)	0
Clostridia	Clostridium difficile P37 (uid86013)	0
Clostridia	Clostridium difficile P38 (uid86007)	0
Clostridia	Clostridium difficile P41 (uid86009)	0

Clostridia	Clostridium difficile P42 (uid86015)	0
Clostridia	Clostridium difficile P45 (uid86017)	0
Clostridia	Clostridium difficile P46 (uid86019)	0
Clostridia	Clostridium difficile P48 (uid86021)	0
Clostridia	Clostridium difficile P49 (uid86023)	0
Clostridia	Clostridium difficile P5 (uid85967)	0
Clostridia	Clostridium difficile P51 (uid86027)	0
Clostridia	Clostridium difficile P59 (uid86033)	0
Clostridia	Clostridium difficile P6 (uid85969)	0
Clostridia	Clostridium difficile P61 (uid86035)	0
Clostridia	Clostridium difficile P68 (uid86041)	0
Clostridia	Clostridium difficile P69 (uid86043)	0
Clostridia	Clostridium difficile P7 (uid85971)	0
Clostridia	Clostridium difficile P71 (uid86049)	0
Clostridia	Clostridium difficile P72 (uid86047)	0
Clostridia	Clostridium difficile P74 (uid86053)	0
Clostridia	Clostridium difficile P75 (uid86055)	0
Clostridia	Clostridium difficile P77 (uid86057)	0
Clostridia	Clostridium difficile P78 (uid86037)	0
Clostridia	Clostridium difficile P8 (uid85973)	0
Clostridia	Clostridium difficile P9 (uid85975)	0
Clostridia	Clostridium difficile R20291 (uid38039)	0
Clostridia	Clostridium difficile T10 (uid222753)	0
Clostridia	Clostridium difficile T11 (uid222743)	0
Clostridia	Clostridium difficile T14 (uid222739)	0
Clostridia	Clostridium difficile T15 (uid222740)	0
Clostridia	Clostridium difficile T17 (uid222730)	0
Clostridia	Clostridium difficile T19 (uid222749)	0
Clostridia	Clostridium difficile T20 (uid222735)	0
Clostridia	Clostridium difficile T23 (uid222737)	0
Clostridia	Clostridium difficile T42 (uid222747)	0
Clostridia	Clostridium difficile T5 (uid222741)	0
Clostridia	Clostridium difficile T6 (uid222751)	0
Clostridia	Clostridium difficile T61 (uid222732)	0
Clostridia	Clostridium difficile Y155 (uid85927)	0
Clostridia	Clostridium difficile Y165 (uid85929)	0
Clostridia	Clostridium difficile Y171 (uid85931)	0
Clostridia	Clostridium difficile Y184 (uid85933)	0
Clostridia	Clostridium difficile Y202 (uid85935)	0
Clostridia	Clostridium difficile Y21 (uid85923)	0
Clostridia	Clostridium difficile Y215 (uid85937)	0
Clostridia	Clostridium difficile Y231 (uid85939)	0
Clostridia	Clostridium difficile Y247 (uid85941)	0
Clostridia	Clostridium difficile Y266 (uid85943)	0
Clostridia	Clostridium difficile Y307 (uid85947)	0
Clostridia	Clostridium difficile Y312 (uid85949)	0
Clostridia	Clostridium difficile Y343 (uid85951)	0
Clostridia	Clostridium difficile Y358 (uid85953)	0
Clostridia	Clostridium difficile Y381 (uid85955)	0
Clostridia	Clostridium difficile Y384 (uid85957)	0
Clostridia	Clostridium difficile Y401 (uid85959)	0
Clostridia	Clostridium difficile Y41 (uid85925)	0
Clostridia	Clostridium DL VIII (uid42093)	0
Clostridia	Clostridium hathewayi 12489931 (uid64875)	0
Clostridia	Clostridium hathewayi DSM 13479 (uid30755)	0
Clostridia	Clostridium hathewayi WAL 18680 (uid46393)	0
Clostridia	Clostridium HGF2 (uid54027)	0
Clostridia	Clostridium hiranonis DSM 13275 (uid28657)	0
Clostridia	Clostridium kluyveri DSM 555 (uid19065)	0
Clostridia	Clostridium kluyveri NBRC 12016 (uid33271)	0
Clostridia	Clostridium lentocellum DSM 5427 (uid41511)	0
Clostridia	Clostridium leptum DSM 753 (uid18923)	0
Clostridia	Clostridium ljungdahlii ATCC 49587 (uid13492)	0
Clostridia	Clostridium M62 1 (uid18205)	0
Clostridia	Clostridium methylpentosum DSM 5476 (uid30029)	0
Clostridia	Clostridium MSTE9 (uid78561)	0
Clostridia	Clostridium nexile DSM 1787 (uid28659)	0
Clostridia	Clostridium novyi NT (uid16820)	0
Clostridia	Clostridium papyrosolvens C7 (uid201398)	0
Clostridia	Clostridium papyrosolvens DSM 2782 (uid33587)	0
Clostridia	Clostridium pasteurianum BC1 (uid80)	0
Clostridia	Clostridium pasteurianum DSM 525 (uid182067)	0
Clostridia	Clostridium pasteurianum NRRL B 598 (uid229510)	0
Clostridia	Clostridium perfringens (uid79)	0
Clostridia	Clostridium perfringens ATCC 13124 (uid304)	0
Clostridia	Clostridium perfringens B ATCC 3626 (uid20027)	0

Clostridia	Clostridium perfringens C JGS1495 (uid20025)	0	
Clostridia	Clostridium perfringens CPE F4969 (uid20031)	0	
Clostridia	Clostridium perfringens D JGS1721 (uid28587)	0	
Clostridia	Clostridium perfringens E JGS1987 (uid20029)	0	
Clostridia	Clostridium perfringens F262 (uid51969)	0	
Clostridia	Clostridium perfringens NCTC 8239 (uid20033)	0	
Clostridia	Clostridium perfringens SM101 (uid12521)	0	
Clostridia	Clostridium perfringens WAL 14572 (uid46397)	0	
Clostridia	Clostridium phytofermentans ISDg (uid16184)	0	
Clostridia	Clostridium saccharobutylicum DSM 13864 (uid217481)	0	
Clostridia	Clostridium saccharolyticum WM1 (uid42097)	0	
Clostridia	Clostridium saccharolyticum)	0	
Clostridia	Clostridium saccharoperbutylacetonicum ATCC 27021 (uid60605)	0	
Clostridia	Clostridium sartagoforme AAU1 buffalo (uid168248)	0	
Clostridia	Clostridium scindens ATCC 35704 (uid18175)	0	
Clostridia	Clostridium sordellii VPI 9048 (uid188694)	0	
Clostridia	Clostridium sp. ASBs410)	0	
Clostridia	Clostridium sp. ASF502)	0	
Clostridia	Clostridium sp. KLE 1755)	0	Clostridiales;Lachnospiraceae;Eisenbergiella
Clostridia	Clostridium sporogenes PA3679 (uid71727)	0	
Clostridia	Clostridium SS2 1 (uid18201)	0	
Clostridia	Clostridium stercorarium DSM 8532 (uid181307)	0	
Clostridia	Clostridium sticklandii DSM 519 (uid28575)	0	
Clostridia	Clostridium straminisolvens JCM 21531 (uid235992)	0	
Clostridia	Clostridium SY8519 (uid67799)	0	
Clostridia	Clostridium symbiosum WAL 14163 (uid46399)	0	
Clostridia	Clostridium symbiosum WAL 14673 (uid46401)	0	
Clostridia	Clostridium termitidis CT1112 (uid167606)	0	
Clostridia	Clostridium tetani E88 (uid81)	0	
Clostridia	Clostridium tetanomorphum DSM 665 (uid174458)	0	
Clostridia	Clostridium thermocellum AD2 (uid82183)	0	
Clostridia	Clostridium thermocellum BC1 (uid223245)	0	
Clostridia	Clostridium thermocellum DSM 1313 (uid41553)	0	
Clostridia	Clostridium thermocellum DSM 2360 (uid32579)	0	
Clostridia	Clostridium thermocellum JW20 (uid28257)	0	
Clostridia	Clostridium thermocellum YS (uid73891)	0	
Clostridia	Clostridium tyrobutyricum DIVETGP (uid231576)	0	
Clostridia	Clostridium ultunense Esp (uid189739)	0	
Clostridia	Coprococcus ART55 1 (uid45915)	0	
Clostridia	Coprococcus catus GD 7 (uid45861)	0	
Clostridia	Coprococcus comes ATCC 27758 (uid20525)	0	
Clostridia	Coprococcus eutactus ATCC 27759 (uid18187)	0	
Clostridia	Coprococcus HPP0048 (uid72501)	0	
Clostridia	Coprococcus HPP0074 (uid72499)	0	
Clostridia	Coprococcus sp. HPP0048)	0	
Clostridia	Coprococcus sp. HPP0074)	0	
Clostridia	Coprothermobacter proteolyticus DSM 5265 (uid30729)	0	
Actinobacteria	Corynebacterium glutamicum ATCC 13032)	0	
Clostridia	Dehalobacter 11DCA (uid83115)	0	
Clostridia	Dehalobacter CF (uid80805)	0	
Clostridia	Dehalobacter restrictus DSM 9455 (uid66209)	0	
Clostridia	Dehalobacter TCM (uid205884)	0	
Clostridia	Desulfitobacterium dehalogenans ATCC 51507 (uid61181)	0	
Clostridia	Desulfitobacterium dichloroeliminans LMG P 21439 (uid61519)	0	
Clostridia	Desulfitobacterium hafniense DCB 2 (uid205)	0	
Clostridia	Desulfitobacterium hafniense Y51 (uid16639)	0	
Clostridia	Desulfosporosinus acidiphilus SJ4 (uid61821)	0	
Clostridia	Desulfosporosinus meridiei DSM 13257 (uid62669)	0	
Clostridia	Desulfosporosinus orientis DSM 765 (uid66191)	0	
Clostridia	Desulfosporosinus OT (uid61253)	0	
Clostridia	Desulfosporosinus youngiae DSM 17734 (uid60451)	0	
Clostridia	Desulfotomaculum acetoxidans DSM 771 (uid27947)	0	
Clostridia	Desulfotomaculum carboxydivorans CO 1 SRB (uid50757)	0	
Clostridia	Desulfotomaculum hydrothermale Lam5 DSM 18033 (uid179138)	0	
Clostridia	Desulfotomaculum kuznetsovii DSM 6115 (uid48313)	0	
Clostridia	Desulfotomaculum reducens MI-1 (uid13424)	0	
Clostridia	Desulfotomaculum ruminis DSM 2154 (uid47605)	0	
Clostridia	Dethiobacter alkaliphilus AHT 1 (uid30985)	0	
Clostridia	Dorea 41031 (uid175992)	0	
Clostridia	Dorea formicigenerans 4 6 53AFAA (uid46407)	0	
Clostridia	Dorea formicigenerans ATCC 27755 (uid18155)	0	
Proteobacteria	Escherichia coli str. K-12 substr. MG1655)	0	
Clostridia	Ethanoligenens harbinense YUAN 3 (uid39729)	0	
Clostridia	Eubacteriaceae bacterium ACC19a (uid49887)	0	
Clostridia	Eubacteriaceae bacterium CM5 (uid49891)	0	
Clostridia	Eubacteriaceae bacterium OBRC8 (uid78565)	0	

Clostridia	Eubacterium 14 2 (uid175980)	0
Clostridia	Eubacterium 3 1 31 (uid39375)	0
Clostridia	Eubacterium AS15 (uid78563)	0
Clostridia	Eubacterium brachy ATCC 33089 (uid198877)	0
Clostridia	Eubacterium cellulosolvens 6 (uid45821)	0
Clostridia	Eubacterium eligens ATCC 27750 (uid29073)	0
Clostridia	Eubacterium eligens)	0
Clostridia	Eubacterium hadrum DSM 3319 (uid38323)	0
Clostridia	Eubacterium hallii DSM 3353 (uid18177)	0
Clostridia	Eubacterium infirmum F0142 (uid52085)	0
Clostridia	Eubacterium limosum KIST612 (uid52281)	0
Clostridia	Eubacterium plexicaudatum ASF492 (uid176002)	0
Clostridia	Eubacterium plexicaudatum ASF492)	0
Clostridia	Eubacterium rectale (uid39161)	0
Clostridia	Eubacterium rectale ATCC 33656 (uid29071)	0
Clostridia	Eubacterium saburreum DSM 3986 (uid52981)	0
Clostridia	Eubacterium saburreum F0468 (uid75151)	0
Clostridia	Eubacterium saphenum ATCC 49989 (uid33149)	0
Clostridia	Eubacterium siraeum (uid39157)	0
Clostridia	Eubacterium siraeum DSM 15702 (uid18921)	0
Clostridia	Eubacterium siraeum V10Sc8a (uid45919)	0
Clostridia	Eubacterium sp. 14-2)	0
Clostridia	Eubacterium sulci ATCC 35585 (uid53047)	0
Clostridia	Eubacterium ventriosum ATCC 27560 (uid18159)	0
Clostridia	Eubacterium yurii margaretiae ATCC 43715 (uid50511)	0
Clostridia	Faecalibacterium cf prausnitzii KLE1255 (uid46809)	0
Clostridia	Faecalibacterium prausnitzii (uid39151)	0
Clostridia	Faecalibacterium prausnitzii A2 165 (uid18199)	0
Clostridia	Faecalibacterium prausnitzii L2 6 (uid45961)	0
Clostridia	Faecalibacterium prausnitzii M21 2 (uid18203)	0
Clostridia	Fervidicella metallireducens AeB (uid221129)	0
Clostridia	Filifactor alocis ATCC 35896 (uid30485)	0
Clostridia	Finegoldia magna ACS 171 V Col3 (uid49435)	0
Clostridia	Finegoldia magna ALB8 (uid237489)	0
Clostridia	Finegoldia magna ATCC 29328 (uid18981)	0
Clostridia	Finegoldia magna ATCC 53516 (uid31469)	0
Clostridia	Finegoldia magna BVS033A4 (uid51069)	0
Clostridia	Flavonifractor plautii ATCC 29863 (uid18189)	0
Clostridia	Halanaerobium sapolanicus (uid43059)	0
Clostridia	Halanaerobium praevalens DSM 2228 (uid32591)	0
Clostridia	Halobacteroides halobius DSM 5150 (uid46811)	0
Clostridia	Halothermothrix orenii H 168 (uid16377)	0
Clostridia	Helcococcus kunzii ATCC 51366 (uid52183)	0
Clostridia	Heliobacterium modesticaldum Ice1 (uid13427)	0
Clostridia	Johnsonella ignava ATCC 51276 (uid40693)	0
Clostridia	Lachnoanaerobaculum saburreum DSM 3986)	0
Clostridia	Lachnoanaerobaculum saburreum F0468)	0
Clostridia	Lachnospiraceae bacterium 1 1 57FAA (uid39377)	0
Clostridia	Lachnospiraceae bacterium 1 4 56FAA (uid39379)	0
Clostridia	Lachnospiraceae bacterium 10 1 (uid175994)	0
Clostridia	Lachnospiraceae bacterium 10-1)	0
Clostridia	Lachnospiraceae bacterium 2 1 58FAA (uid39381)	0
Clostridia	Lachnospiraceae bacterium 28 4 (uid175982)	0
Clostridia	Lachnospiraceae bacterium 28-4)	0
Clostridia	Lachnospiraceae bacterium 3 1 (uid175981)	0
Clostridia	Lachnospiraceae bacterium 3 1 46FAA (uid40027)	0
Clostridia	Lachnospiraceae bacterium 3-1)	0
Clostridia	Lachnospiraceae bacterium 4 1 37FAA (uid41967)	0
Clostridia	Lachnospiraceae bacterium 4 1 37FAA)	0
Clostridia	Lachnospiraceae bacterium 40970 (uid175993)	0
Clostridia	Lachnospiraceae bacterium 5 1 57FAA (uid39387)	0
Clostridia	Lachnospiraceae bacterium 5 1 63FAA (uid39389)	0
Clostridia	Lachnospiraceae bacterium 6 1 63FAA (uid39395)	0
Clostridia	Lachnospiraceae bacterium 7 1 58FAA (uid39397)	0
Clostridia	Lachnospiraceae bacterium 8 1 57FAA (uid40029)	0
Clostridia	Lachnospiraceae bacterium 9 1 43BFAA (uid39399)	0
Clostridia	Lachnospiraceae bacterium A2 (uid175985)	0
Clostridia	Lachnospiraceae bacterium A2)	0
Clostridia	Lachnospiraceae bacterium A4)	0
Clostridia	Lachnospiraceae bacterium ACC2 (uid49875)	0
Clostridia	Lachnospiraceae bacterium COE1 (uid175984)	0
Clostridia	Lachnospiraceae bacterium COE1)	0
Clostridia	Lachnospiraceae bacterium JC7 (uid109283)	0
Clostridia	Lachnospiraceae bacterium M18 1 (uid175983)	0
Clostridia	Lachnospiraceae bacterium M18-1)	0
Clostridia	Lachnospiraceae bacterium MSX33 (uid165899)	0

Clostridia	Lachnospiraceae bacterium oral taxon 082 F0431 (uid50401)	0
Clostridia	Lachnospiraceae oral taxon 107 F0167 (uid42531)	0
Bacilli	Lactobacillus plantarum WCFS1)	0
Clostridia	Mahella australiensis 50 1 BON (uid42243)	0
Clostridia	Marvinbryantia formatexigens DSM 14469)	0
Clostridia	Mogibacterium CM50 (uid78583)	0
Clostridia	Mogibacterium timidum ATCC 33093 (uid219678)	0
Clostridia	Moorella thermoacetica ATCC 39073 (uid10648)	0
Actinobacteria	Mycobacterium tuberculosis H37Rv)	0
Clostridia	Natranaerobius thermophilus JW NM WN LF (uid20207)	0
Clostridia	Oribacterium ACB1 (uid49877)	0
Clostridia	Oribacterium ACB8 (uid78569)	0
Clostridia	Oribacterium oral taxon 078 F0262 (uid33167)	0
Clostridia	Oribacterium oral taxon 078 F0263 (uid198889)	0
Clostridia	Oribacterium oral taxon 108 F0425 (uid53571)	0
Clostridia	Oribacterium sp. oral taxon 078 str. F0263)	0
Clostridia	Oscillibacter 40911 (uid175991)	0
Clostridia	Oscillibacter sp. 1-3)	0
Clostridia	Parvimonas micra ATCC 33270 (uid18169)	0
Clostridia	Parvimonas oral taxon 110 F0139 (uid49295)	0
Clostridia	Parvimonas oral taxon 393 F0440 (uid61837)	0
Clostridia	Pelotomaculum thermopropionicum SI (uid19023)	0
Clostridia	Peptococcaceae bacterium SCADC (uid243645)	0
Clostridia	Peptoniphilus harei ACS 146 V Sch2b (uid54023)	0
Clostridia	Peptoniphilus indolicus ATCC 29427 (uid64733)	0
Clostridia	Peptoniphilus lacrimalis 315 B (uid34107)	0
Clostridia	Peptoniphilus oral taxon 375 F0436 (uid52051)	0
Clostridia	Peptoniphilus oral taxon 836 F0141 (uid49437)	0
Clostridia	Peptostreptococcus anaerobius 653 L (uid34105)	0
Clostridia	Peptostreptococcus anaerobius VPI 4330 (uid67203)	0
Clostridia	Peptostreptococcus stomatis DSM 17678 (uid34073)	0
Clostridia	Pseudoflavonifractor capillosus ATCC 29799)	0
Proteobacteria	Pseudomonas aeruginosa PAO1-VE13)	0
Clostridia	Roseburia hominis A2 183 (uid33399)	0
Clostridia	Roseburia hominis A2-183)	0
Clostridia	Roseburia intestinalis (uid39165)	0
Clostridia	Roseburia intestinalis L1 82 (uid30005)	0
Clostridia	Roseburia intestinalis XB6B4 (uid45953)	0
Clostridia	Roseburia inulinivorans DSM 16841 (uid30757)	0
Clostridia	Ruminococcaceae bacterium D16 (uid42541)	0
Clostridia	Ruminococcaceae bacterium D16)	0
Clostridia	Ruminococcus (uid39149)	0
Clostridia	Ruminococcus 18P13 (uid39179)	0
Clostridia	Ruminococcus 5 1 39BFAA (uid32503)	0
Clostridia	Ruminococcus albus 7 (uid42255)	0
Clostridia	Ruminococcus albus 7 = DSM 20455)	0
Clostridia	Ruminococcus albus 8 (uid62)	0
Clostridia	Ruminococcus albus SY3 (uid206423)	0
Clostridia	Ruminococcus bromii (uid39153)	0
Clostridia	Ruminococcus flavefaciens 007c (uid206425)	0
Clostridia	Ruminococcus gnavus ATCC 29149 (uid18179)	0
Clostridia	Ruminococcus gnavus CC55 001C (uid71557)	0
Clostridia	Ruminococcus lactaris ATCC 29176 (uid20557)	0
Clostridia	Ruminococcus lactaris CC59 002D (uid71573)	0
Clostridia	Ruminococcus torques (uid39169)	0
Clostridia	Ruminococcus torques ATCC 27756 (uid18153)	0
Clostridia	Shuttleworthia MSX8B (uid165923)	0
Clostridia	Shuttleworthia satelles DSM 14600 (uid33169)	0
Bacilli	Staphylococcus aureus subsp. aureus Mu50)	0
Bacilli	Streptococcus pyogenes MGAS315)	0
Clostridia	Subdoligranulum 4 3 54A2FAA (uid40041)	0
Clostridia	Subdoligranulum variabile DSM 15176 (uid18181)	0
Clostridia	Sulfobacillus acidophilus DSM 10332 (uid40777)	0
Clostridia	Sulfobacillus acidophilus TPY (uid68423)	0
Clostridia	Symbiobacterium thermophilum IAM14863 (uid12994)	0
Clostridia	Syntrophobotulus glycolicus DSM 8271 (uid38111)	0
Clostridia	Syntrophomonas wolfei Goettingen (uid13014)	0
Clostridia	Syntrophothermus lipocalidus DSM 12680 (uid37873)	0
Clostridia	Tepidanaerobacter acetatoxydans Re1 (uid184009)	0
Clostridia	Tepidanaerobacter Re1 (uid50697)	0
Clostridia	Thermacetogenium phaeum DSM 12270 (uid168373)	0
Clostridia	Thermaerobacter subterraneus DSM 13965 (uid50747)	0
Clostridia	Thermoanaerobacter brockii finnii Ako 1 (uid32585)	0
Clostridia	Thermoanaerobacter ethanolicus CCSD1 (uid33633)	0
Clostridia	Thermoanaerobacter ethanolicus JW 200 (uid51151)	0
Clostridia	Thermoanaerobacter italicus Ab9 (uid33157)	0

Clostridia	Thermoanaerobacter mathranii A3 (uid33329)	0
Clostridia	Thermoanaerobacter pseudethanolicus ATCC 33223 (uid13901)	0
Clostridia	Thermoanaerobacter siderophilus SR4 (uid64483)	0
Clostridia	Thermoanaerobacter tengcongensis (uid249)	0
Clostridia	Thermoanaerobacter thermohydrosulfuricus WC1 (uid168425)	0
Clostridia	Thermoanaerobacter X513 (uid32613)	0
Clostridia	Thermoanaerobacter X561 (uid33613)	0
Clostridia	Thermoanaerobacterium aotearoense SCUT27 (uid227407)	0
Clostridia	Thermoanaerobacterium saccharolyticum JW SL YS485 (uid73961)	0
Clostridia	Thermoanaerobacterium thermosaccharolyticum DSM 571 (uid33165)	0
Clostridia	Thermoanaerobacterium thermosaccharolyticum M0795 (uid60809)	0
Clostridia	Thermoanaerobacterium xylanolyticum LX 11 (uid50295)	0
Clostridia	Thermosediminibacter oceani DSM 16646 (uid30983)	0
Clostridia	Tropheryma whipplei Art1 (uid189644)	0
Clostridia	Youngiibacter fragile 232 1 (uid223318)	0

Table S2. The details of the GO enhancement test in Figure 4. Each data point in Figure 4 represents one GO term.

Height = 80

	GOterm	pvalue	adjustedP	statistics	GOfreq
GO:0000160	phosphorelay signal transduction system	0	0	136.125	160
GO:0006935	chemotaxis	0	0	99.18431	19
GO:0008643	carbohydrate transport	0	0	86.17984	199
GO:0009236	cobalamin biosynthetic process	0	0	198	17
GO:0071973	bacterial-type flagellum-dependent cell motility	0	0	79.09893	28
GO:0006810	transport	0.00522	0.00696	28.57122	157
GO:0009306	protein secretion	0.00991	0.011325714	41.03627	12
GO:0044780	bacterial-type flagellum assembly	0.01546	0.01546	35.57813	12

Height = 84

	GOterm	pvalue	adjustedP	statistics	GOfreq
GO:0000160	phosphorelay signal transduction system	0	0	143.2578	160
GO:0005975	carbohydrate metabolic process	0	0	273	211
GO:0006351	transcription, DNA-templated	0	0	124.304	417
GO:0006935	chemotaxis	0	0	134.8065	19
GO:0008643	carbohydrate transport	0	0	123.678	199
GO:0009236	cobalamin biosynthetic process	0	0	273	17
GO:0006810	transport	1.00E-05	1.25E-05	55.37315	157
GO:0071973	bacterial-type flagellum-dependent cell motility	1.00E-05	1.25E-05	102.5719	28
GO:0044780	bacterial-type flagellum assembly	0.01486	0.016511111	53.40899	12
GO:0009306	protein secretion	0.03383	0.03383	48.66239	12

Height = 88

	GOterm	pvalue	adjustedP	statistics	GOfreq
GO:0000160	phosphorelay signal transduction system	0	0	160.8456	160
GO:0005975	carbohydrate metabolic process	0	0	286.1506	211
GO:0006351	transcription, DNA-templated	0	0	172.2928	417
GO:0006810	transport	0	0	86.19056	157
GO:0008643	carbohydrate transport	0	0	180.2221	199
GO:0009236	cobalamin biosynthetic process	0	0	359	17
GO:0006935	chemotaxis	1.00E-05	1.86E-05	150.1072	19
GO:0006310	DNA recombination	2.00E-05	2.89E-05	196.7325	28
GO:0071973	bacterial-type flagellum-dependent cell motility	2.00E-05	2.89E-05	136.4447	28
GO:0015074	DNA integration	0.00039	0.000507	164.829	26
GO:0006355	regulation of transcription, DNA-templated	0.0074	0.008745455	110.2184	146
GO:0044780	bacterial-type flagellum assembly	0.03802	0.041188333	67.92455	12
GO:0009306	protein secretion	0.09756	0.09756	60.2802	12

Height = 92

	GOterm	pvalue	adjustedP	statistics	GOfreq
GO:0000160	phosphorelay signal transduction system	0	0	187.9679	160
GO:0005975	carbohydrate metabolic process	0	0	267.6674	211
GO:0006351	transcription, DNA-templated	0	0	204.9879	417
GO:0006412	translation	0	0	366.9903	59
GO:0006526	arginine biosynthetic process	0	0	441	8
GO:0006810	transport	0	0	121.3031	157
GO:0006935	chemotaxis	0	0	185.7468	19
GO:0008643	carbohydrate transport	0	0	229.9255	199
GO:0009236	cobalamin biosynthetic process	0	0	441	17
GO:0006355	regulation of transcription, DNA-templated	1.00E-05	1.60E-05	204.1032	146
GO:0071973	bacterial-type flagellum-dependent cell motility	4.00E-05	5.82E-05	153.4102	28

GO:0006310	DNA recombination	0.00105	0.0014	170.7097	28
GO:0006352	DNA-templated transcription, initiation	0.0042	0.005169231	180.3043	34
GO:0015074	DNA integration	0.00459	0.005245714	175.8437	26
GO:0044780	bacterial-type flagellum assembly	0.09442	0.100714667	81.12032	12
GO:0009306	protein secretion	0.14799	0.14799	74.64052	12

Height = 96

	GOterm	pvalue	adjustedP	statistics	GOfreq
GO:0000160	phosphorelay signal transduction system	0	0	239.8334	160
GO:0005975	carbohydrate metabolic process	0	0	321.1122	211
GO:0006013	mannose metabolic process	0	0	559.6146	17
GO:0006351	transcription, DNA-templated	0	0	252.842	417
GO:0006412	translation	0	0	601.4885	59
GO:0006810	transport	0	0	211.6311	157
GO:0008643	carbohydrate transport	0	0	371.8874	199
GO:0009236	cobalamin biosynthetic process	0	0	575.6635	17
GO:0015833	peptide transport	0	0	519.7587	19
GO:0042121	alginic acid biosynthetic process	0	0	686	7
GO:0071973	bacterial-type flagellum-dependent cell motility	4.00E-05	9.09E-05	226.8002	28
GO:0006355	regulation of transcription, DNA-templated	5.00E-05	0.000104167	207.7765	146
GO:0006526	arginine biosynthetic process	9.00E-05	0.000173077	374.5762	8
GO:0006352	DNA-templated transcription, initiation	0.00028	5.00E-04	297.2881	34
GO:0006935	chemotaxis	8.00E-04	0.001333333	210.879	19
GO:0007049	cell cycle	0.00506	0.00790625	257.3117	16
GO:0051301	cell division	0.00667	0.009808824	233.1457	19
GO:0000105	histidine biosynthetic process	0.0073	0.010138889	244.7289	10
GO:0006260	DNA replication	0.01058	0.013921053	245.0528	29
GO:0006281	DNA repair	0.01209	0.0151125	241.6988	26
GO:0006310	DNA recombination	0.03989	0.047488095	168.0331	28
GO:0015074	DNA integration	0.04652	0.052863636	179.1599	26
GO:0055085	transmembrane transport	0.08975	0.097554348	161.1412	38
GO:0009306	protein secretion	0.16901	0.176052083	120.0461	12
GO:0044780	bacterial-type flagellum assembly	0.18922	0.18922	113.8631	12

Height = 100

	GOterm	pvalue	adjustedP	statistics	GOfreq
GO:0005975	carbohydrate metabolic process	0	0	333.8726	211
GO:0005978	glycogen biosynthetic process	0	0	1135	6
GO:0006004	fucose metabolic process	0	0	708.3949	14
GO:0006351	transcription, DNA-templated	0	0	197.3414	417
GO:0006355	regulation of transcription, DNA-templated	0	0	283.5487	146
GO:0006412	translation	0	0	590.478	59
GO:0006810	transport	0	0	258.5958	157
GO:0008643	carbohydrate transport	0	0	524.2886	199
GO:0009236	cobalamin biosynthetic process	0	0	576.6328	17
GO:0015833	peptide transport	0	0	497.7065	19
GO:0006013	mannose metabolic process	1.00E-05	4.45E-05	449.0719	17
GO:0006260	DNA replication	4.00E-05	0.000163333	373.4774	29
GO:0006457	protein folding	0.00057	0.002148462	483.9697	13
GO:0006935	chemotaxis	0.00107	0.003745	299.6739	19
GO:0042121	alginic acid biosynthetic process	0.00118	0.003854667	450.3809	7
GO:0000160	phosphorelay signal transduction system	0.00306	0.00937125	172.8913	160
GO:0055085	transmembrane transport	0.00439	0.012653529	268.8528	38
GO:0006352	DNA-templated transcription, initiation	0.00521	0.014182778	249.4661	34
GO:0006189	'de novo' IMP biosynthetic process	0.00582	0.014259	332.1078	11
GO:0044205	'de novo' UMP biosynthetic process	0.00565	0.014259	354.8378	7
GO:0008360	regulation of cell shape	0.00716	0.016706667	358.4644	13

GO:0006779	porphyrin-containing compound biosynthetic process	0.00848	0.018343043	349.9437	24
GO:0071973	bacterial-type flagellum-dependent cell motility	0.00861	0.018343043	223.9585	28
GO:0006265	DNA topological change	0.01032	0.02107	321.0849	12
GO:0006541	glutamine metabolic process	0.01292	0.0253232	329.3623	9
GO:0009098	leucine biosynthetic process	0.0198	0.037315385	333.5299	5
GO:0006520	cellular amino acid metabolic process	0.02791	0.050651481	315.0132	7
GO:0009058	biosynthetic process	0.0388	0.0679	237.9085	20
GO:0000105	histidine biosynthetic process	0.04078	0.068904138	245.6203	10
GO:0007049	cell cycle	0.05211	0.084943871	218.1892	16
GO:0009253	peptidoglycan catabolic process	0.05374	0.084943871	275.2333	8
GO:0006310	DNA recombination	0.06248	0.094436364	184.4889	28
GO:0006364	rRNA processing	0.0636	0.094436364	245.0927	9
GO:0006814	sodium ion transport	0.06698	0.09653	258.0599	9
GO:0006526	arginine biosynthetic process	0.07016	0.098224	239.6448	8
GO:0030435	sporulation resulting in formation of a cellular spore	0.09025	0.120063243	232.5825	8
GO:0046355	mannan catabolic process	0.09066	0.120063243	232.4825	7
GO:0000917	barrier septum assembly	0.0973	0.125465789	215.8354	9
GO:0051301	cell division	0.10092	0.126796923	182.1395	19
GO:0009306	protein secretion	0.11082	0.13435561	184.917	12
GO:0009401	phosphoenolpyruvate-dependent sugar phosphotransferase system	0.11242	0.13435561	206.6963	8
GO:0046373	L-arabinose metabolic process	0.16133	0.188218333	172.3192	11
GO:0006281	DNA repair	0.1765	0.201127907	156.672	26
GO:0015074	DNA integration	0.19389	0.215922955	153.8093	26
GO:0009432	SOS response	0.22821	0.248495333	150.0501	9
GO:0044780	bacterial-type flagellum assembly	0.25833	0.275177609	140.1359	12
GO:0006396	RNA processing	0.32394	0.337724681	126.7068	10
GO:0045892	negative regulation of transcription, DNA-templated	0.4507	0.460089583	105.1301	17
GO:0006012	galactose metabolic process	0.53811	0.53811	88.86893	9

Table S3. Phylogenetic affinities of LZ in 35 phylogenetic trees of proteins from the flagellar assembly / motility cluster. Proteins are sorted in increasing order of GenBank GI number. Asterisks indicate trees that contain more than 1 representative from LZ; each protein is however represented only once in the table. Abbreviations: Cbol7 = *Clostridium bolteae 90B7* ; Cbol8 = *Clostridium bolteae 90B8* ; Cbac28 = *Clostridiales bacterium VE202-28* ; Cbac47 = *Clostridiales bacterium 1_7_47FAA* ; Chyl1 = *Clostridium hylemonae DSM15053* . Clans that comprise only LZ and the other listed genomes are shown; if the genomes do not constitute a clan with LZ, other organisms in the clan are also indicated.

			Present in tree						
Tree #	NCBI GI	Tree size	Cbol7	Cbol8	Cbac28	Cbac47	Chyl1	Present as clan?	Other taxa in clan
1	496544394	57	X	X	X	X		Yes	
15	496544397	60*	X	X	X	X		Yes	
2	496544402	30*	X	X	X	X		Yes	
3	496544405	28	X	X	X	X		Yes	
4	496544418	110	X	X	X	X		Yes	
5	496544427	33*	X	X	X	X		Yes	
6	496544428	64*	X	X	X	X		Yes	
7	496544430	68*	X	X	X	X		Yes	
8	496544432	60*	X	X	X	X		Yes	
9	496544447	75*					X	Yes	
10	496544449	50*					X	Yes	
11	496544470	49*					X	Yes	
5	496544473	33*					X	Yes	
12	496544474	62					X	Yes	
13	496544475	58*					X	No	Oribacterium x 2,C. clostridioforme,Cbac28,Clostridium 7_3_54FAA
14	496544476	38					X	Yes	
15	496544478	60*					X	Yes	
16	496544480	35					X	Yes	
17	496544481	54					X	Yes	
18	496544482	78					X	No	Oribacterium x 2,C. clostridioforme,Cbac28,Clostridium 7_3_54FAA
19	496544485	38					X	Yes	
20	496544487	55					X	Yes	
21	496544494	60*					X	Yes	
22	496544508	191*	X	X				Yes	
26	496546557	387*	X	X	X	X	X	No	C. clostridioforme
23	496547030	178*						No	LZ,Clostridium sp.ASF356
24	496547489	198*	X	X	X	X	X	No	Clostridium 7_3_54FAA
25	496548263	162*			X	X		Yes	
26	496548264	387*					X	Yes	
32	496548406	170*						No	Clostridium sp. ASF356
27	496548609	56	X	X	X	X		Yes	
28	496548610	50	X	X	X	X		Yes	
29	496548611	176*	X	X	X	X		Yes	
30	496548612	69*	X	X	X	X		Yes	
31	496548613	51	X	X	X	X		Yes	
32	496549450	170*	X	X	X			Yes	
33	511537750	42	X	X	X	X		Yes	
34	511537752	57					X	Yes	
35	511538067	70	X	X		X		Yes	

Table S4. The correlations between input distances and dendrogrammatic distances of three hierarchical clustering dendrograms. These three hierarchies are built by running Pagel's program three times on one same small tree (20 genomes).

		Input Distance			Dendrogrammatic Distance		
		1	2	3	1	2	3
Input Distance	1		0.970067	0.970589	0.402408	0.396278	0.386381
	2			0.970498	0.399307	0.399908	0.386841
	3				0.39913	0.396498	0.389219
Dendrogrammatic Distance	1					0.586341	0.574592
	2						0.558288
	3						

Table S5. The average size of clusters (including singletons), weighted mean GO score, number of clusters (excluding singletons) and number of singletons at different cut-offs. The highlighted rows are the three comparable points in the shaded area in Figure S6.

CoPAP					
Cutoffs	P-value	Average Size	GO score	Number of singletons	Number of clusters
0	1	2697	0.296	0	1
0.693	0.5	112.375	0.292	23	1
1.386	0.25	17.861	0.293	140	11
2.303	0.1	4.525	0.324	533	63
2.59	0.075	3.788	0.369	637	75
2.996	0.05	2.935	0.381	825	94
3.283	0.0375	2.514	0.411	970	103
3.689	0.025	2.056	0.464	1187	125
4.046	0.0175	1.746	0.476	1414	131
4.382	0.0125	1.49	0.516	1664	146
4.605	0.01	1.39	0.53	1784	156
4.893	0.0075	1.319	0.541	1905	139
5.298	0.005	1.232	0.557	2062	127
5.586	0.00375	1.194	0.568	2143	115
5.991	0.0025	1.137	0.751	2263	110
6.348	0.00175	1.107	0.754	2348	89
6.502	0.0015	1.095	0.771	2377	87
6.908	0.001	1.069	0.787	2451	73
Pagel with hierarchical clustering					
h	Average Size	GO score	Number of Singletons	Number of Clusters	
110	2697	0.296	0	1	
107.5	134.85	0.311	2	18	
105	27.52	0.346	9	89	
102.5	11.099	0.403	36	207	
100	5.993	0.449	94	356	
97.5	3.684	0.478	270	462	
95	2.591	0.551	519	522	
92.5	2.007	0.593	827	517	
90	1.682	0.659	1154	449	
89	1.587	0.677	1268	431	
88	1.508	0.693	1394	395	
87	1.439	0.713	1509	365	
86	1.381	0.733	1618	335	
85	1.332	0.745	1722	303	
82.5	1.247	0.791	1917	246	
80	1.192	0.8	2054	209	
77.5	1.154	0.805	2161	177	
75	1.125	0.832	2240	158	
72.5	1.095	0.816	2330	132	
70	1.074	0.817	2398	114	
67.5	1.059	0.823	2451	96	
65	1.045	0.851	2501	80	
62.5	1.031	0.851	2560	56	

60	1.024	0.852	2591	44	
CLIME	1.489	0.615	1496	315	
